# Synthesis, antioxidant properties and neuroprotection of α-phenyl-*tert*-butylnitrone derived *HomoBisNitrones* in in vitro and in vivo ischemia models

**DOI:** 10.1038/s41598-020-70690-y

**Published:** 2020-08-25

**Authors:** Beatriz Chamorro, Daniel Diez-Iriepa, Belén Merás-Sáiz, Mourad Chioua, David García-Vieira, Isabel Iriepa, Dimitra Hadjipavlou-Litina, Francisco López-Muñoz, Ricardo Martínez-Murillo, Daniel Gonzàlez-Nieto, Israel Fernández, José Marco-Contelles, María Jesús Oset-Gasque

**Affiliations:** 1 Department of Biochemistry and Molecular Biology, Faculty of Pharmacy, Complutense University of Madrid, 28040 Madrid, Spain; 2 Faculty of Health, Camilo José Cela University of Madrid (UCJC), Madrid, Spain; 3 Laboratory of Medicinal Chemistry, Institute of Organic Chemistry (CSIC), Juan de la Cierva 3, 28006 Madrid, Spain; 4 Department of Organic Chemistry and Inorganic Chemistry, Alcalá University, 28805 Alcalá de Henares, Madrid, Spain; 5 Institute of Chemical Research Andrés M. del Río, Alcalá University, 28805 Alcalá de Henares, Madrid, Spain; 6 Department of Pharmaceutical Chemistry, School of Pharmacy, Faculty of Health Sciences, Aristotle University of Thessaloniki, 54124 Thessaloniki, Greece; 7 Neuropsychopharmacology Unit, “Hospital 12 de Octubre” Research Institute, Madrid, Spain; 8 Neurovascular Research Group, Department of Translational Neurobiology, Cajal Institute (CSIC), Madrid, Spain; 9 Center for Biomedical Technology (CTB), Universidad Politécnica de Madrid, Madrid, Spain; 10 Biomedical Research Networking Center in Bioengineering Biomaterials and Nanomedicine (CIBER-BBN), Madrid, Spain; 11 Departamento de Química Orgánica I and Centro de Innovación en Química Avanzada (ORFEO-CINQA), Facultad de Ciencias Químicas, Universidad Complutense de Madrid, 28040 Madrid, Spain; 12 Instituto Universitario de Investigación en Neuroquímica, Universidad Complutense de Madrid, 28040 Madrid, Spain

**Keywords:** Drug discovery, Neuroscience, Chemistry

## Abstract

We herein report the synthesis, antioxidant power and neuroprotective properties of nine homo-bis-nitrones **HBNs** **1**–**9 **as alpha-phenyl-*N*-*tert*-butylnitrone (**PBN**) analogues for stroke therapy. In vitro neuroprotection studies of **HBNs** **1**–**9** against Oligomycin A/Rotenone and in an oxygen-glucose-deprivation model of ischemia in human neuroblastoma cell cultures, indicate that (1*Z*,1′*Z*)-1,1′-(1,3-phenylene)bis(*N*-benzylmethanimine oxide) (**HBN6**) is a potent neuroprotective agent that prevents the decrease in neuronal metabolic activity (EC_50_ = 1.24 ± 0.39 μM) as well as necrotic and apoptotic cell death. **HBN6** shows strong hydroxyl radical scavenger power (81%), and capacity to decrease superoxide production in human neuroblastoma cell cultures (maximal activity = 95.8 ± 3.6%), values significantly superior to the neuroprotective and antioxidant properties of the parent **PBN**. The higher neuroprotective ability of **HBN6** has been rationalized by means of Density Functional Theory calculations. Calculated physicochemical and ADME properties confirmed **HBN6** as a hit-agent showing suitable drug-like properties. Finally, the contribution of **HBN6** to brain damage prevention was confirmed in a permanent MCAO setting by assessing infarct volume outcome 48 h after stroke in drug administered experimental animals, which provides evidence of a significant reduction of the brain lesion size and strongly suggests that **HBN6** is a potential neuroprotective agent against stroke.

## Introduction

**Figure 1:**
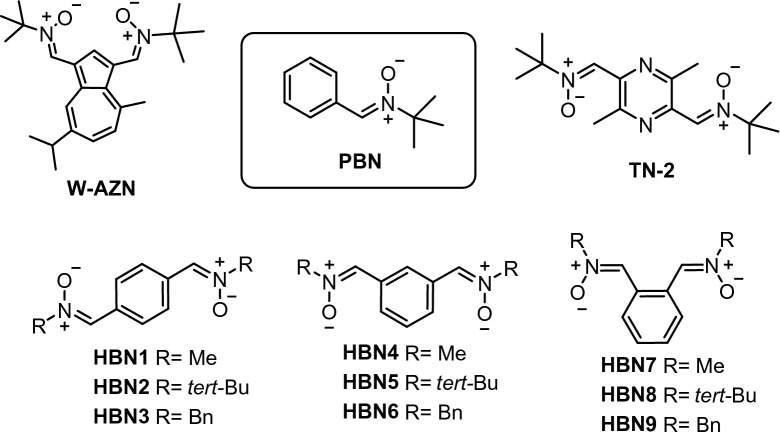
Structures of bis-nitrones W-AZN, PBN, TN-2, and HBNs 1–9.

Bis-nitrones are well-known antioxidant and neuroprotective agents showing high clinical potential. For instance, bis-nitrone **W-AZN** (Fig. 1), an azulenyl spin trap possessing neuroprotective effects in an animal model of cerebral ischemia^1^, is able to attenuate the in vivo MPTP neurotoxicity, suggesting a possible application in the treatment of Parkinson’s disease^2^. Similarly, the neuroprotective capacity of bis-nitrone **STAZN**, a second-generation of potent antioxidant azulenyl nitrone^3^, has been confirmed in focal ischemia models^4,5^. Bis-nitrone **TN-2** (Fig. 1) also exhibits a high neuroprotective effect in either in vitro or in vivo models of stroke, very likely as a consequence of its ability to trap HO^·^ and O_2_^·–^, two of the most toxic reactive oxygen species (ROS) to brain tissues^6,7^.

Despite ROS play key roles in physiological functions at low concentrations, they can also be very toxic in highly oxidative stress dysregulated conditions^8^. For instance, hydroperoxides formed in the reaction of ROS with unsaturated fatty acids are very reactive, and constitute the origin of extensive cell death^9^. Nowadays, it is widely accepted that the formation of ROS is one of the main biological events involved in the etiology of stroke^10^. For this reason, there exists a growing interest in the search for new and more efficient ROS scavengers as potential therapeutic agents for stroke^9^.

In our current program targeted to identify new nitrones for the therapy of stroke^11^, we have already investigated nitrones derived from (hetero)aromatic aldehydes^12,13^, quinolylnitrones^14–17^, and cholesteronitrones^18^. More recently, we have designed bis-nitrones derived from alpha-phenyl-*N*-*tert*-butylnitrone (**PBN**) (Fig. 1), a well-known radical scavenger that prevented and reversed traumatic shock injury in rats^19^, and the starting point of several new nitrones, such as **NXY-059**, the first nitrone to reach clinical trials^20^. As shown in Fig. 1, homo-bis-nitrones (**HBNs**) **1**–**9** result from the incorporation of a second identical nitrone moiety at the *para* (*p-***HBNs 1–3**), *meta* (*m*-**HBNs 4–6**) and *ortho* (*o-***HBNs 7–9**) positions and bearing methyl, *tert*-butyl or benzyl substituents, respectively, as the *N*-alkyl groups attached to the nitrone motif. Among these nitrones, only **HBNs 1**^21^, **2**^22^, **3**^23^, **4**^24,25^, and **5**^26^ have been previously described in the literature, but in studies not related to their antioxidant properties and/or potential use for stroke therapy. In fact, the present work is the first study aimed at exploring the neuroprotective and antioxidant properties of bis-nitrones, analogues of the parent **PBN**. The hypothesis behind the present design is that two “nitrone” scavenging motifs in the same scaffold should afford a higher antioxidant power than only one. Indeed, as it will be shown later on, we have identified (1*Z*,1′*Z*)-1,1′-(1,3-phenylene)bis(*N*-benzylmethanimine oxide) (**HBN6**) as a potent neuroprotective ligand (EC_50_ = 1.24 ± 0.39 µM), whose neuroprotective and antioxidant capacities are higher than those of **PBN**.

## Results and discussion

### Chemistry

**Scheme 1:**
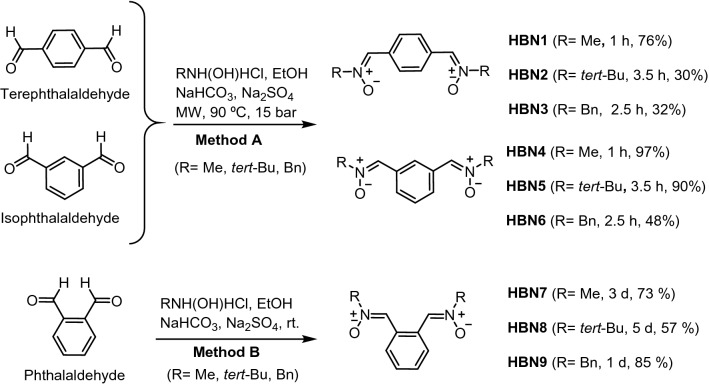
Synthesis of **HBNs 1–9**.

The synthesis of **HBNs 1–9** (Scheme 1) was achieved from commercial and readily available carbaldehyde precursors (terephthalaldehyde, isophthalaldehyde, and phthalaldehyde) and the appropriate *N*-methyl(*tert*-butyl, benzyl)hydroxylamine hydrochlorides (Methods and Supplementary Information). All compounds were isolated as pure *Z* isomers at the double PhC=N(O)R bond, and exhibited analytical and spectroscopic data in agreement to those previously reported^21–26^.

## Neuroprotection studies

### Oligomycin A/rotenone

One of the first events taking place in the initial stages of stroke is the collapse of the mitochondrial electron transport chain (ETC), which leads to extended cell death and brain damage due to the formation of ROS. In order to mimic this event into suitable experiments, we tested the effect of the bis-nitrones on cell death induced by Oligomycin A and Rotenone (O/R), inhibitors of mitochondrial complexes V and I, respectively. To this end, we used the XTT cell viability test, a colorimetric assay that detects the cellular metabolic activities. Based on a previous work from our laboratory^27^, we selected the appropriate experimental conditions and tested the neuroprotective effect of **HBNs 1–9** at different concentrations (0.1–1,000 μM), added 10 min before the administration of O10 μM /R30 μM (O/R), and using **PBN**, at the same concentrations (0.1–1,000 μM), as a reference compound^28^.

**Figure 2:**
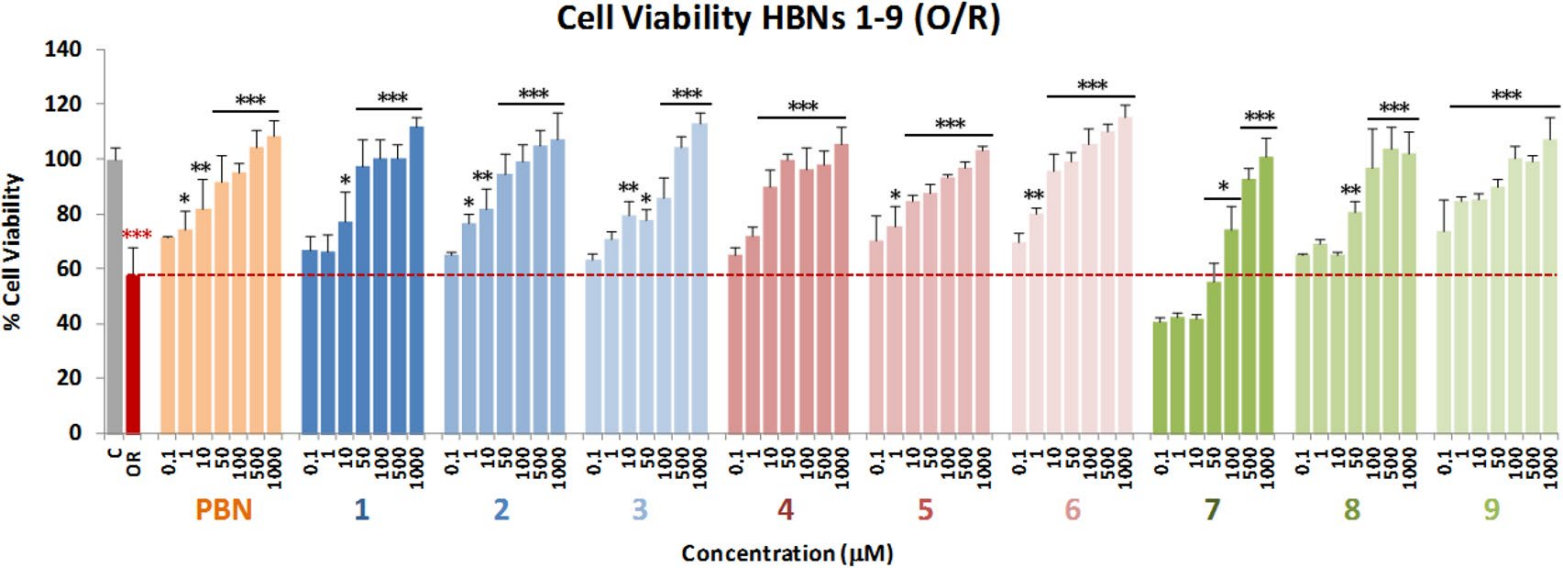
Neuroprotective effect of **HBNs 1–9** on SH-SY5Y human neuroblastoma cells viability after treatment with O/R. Bars show % cell viability after treatment with O10/R30, with, or without, **HBNs 1–9** and **PBN**, at the indicated concentrations. Values are the mean ± SEM of three experiments, each one performed in triplicate. The statistics compare the effect of OR on its control (C) (red ***) or the effect of the different compounds after O/R (24 h) with O/R (24 h) alone, in the absence of these compounds (black ***). Data were statistically analyzed by one-way ANOVA, followed by Holm-Sidak as test post hoc. *P < 0.05; **P < 0.01; ***P < 0.001.

As shown in Fig. 2, a 42.31 ± 4.43% (mean ± SEM) inhibition of neuroblastoma cells viability was observed upon treatment with O10/R30 for 24 h. This effect was reverted after incubation with **PBN** and **HBNs 1–9** for 24 h in a concentration-dependent manner (Fig. 2). The neuroprotection study, considering the 100% neuroprotection as the difference between C24 h viability (100 ± 4.75%; mean ± SEM; n = 20) and OR (57.69 ± 10.46; mean ± SEM; n = 16) revealed that the most potent nitrones were **HBNs 4–6**. Table 1 gathers the analyses of concentration–response curves for **HBNs 1–9** and** PBN**, in the range of 0.1 μM to 1 mM, the corresponding EC_50_ values, and the highest neuroprotective activities. EC_50_ values, from the lowest to the highest, follows the order: **HBN5** ≤ **NAC** ≤** HBN6** ≤ **HBN4** ≤ **HBN3** ≤ **HBN2** << **HBN9** < **HBN8** ≤ **HBN1** ≤ **PBN** <<< **HBN7**.

**Table 1:** Neuroprotective effect of **HBNs 1–9**, and **PBN** after O/R treatment in human neuroblastoma SH-SY5Y cells.

	HBN	R	Neuroprotection (EC_50_ ± SEM), μM	*P* < (PBN)	*P* < (HBN6)	Maximal activity (Mean ± SEM), %	*P* < (PBN)	*P* < (HBN6)
*p*-**HBNs (1–3)**	**1**	Me	62.15 ± 18.18	ns	***	116.13 ± 9.52	ns	ns
	**2**	*tert*-Bu	21.07 ± 5.30	***	ns	129.86 ± 8.81	ns	ns
	**3**	Bn	18.72 ± 3.02	***	ns	126.98 ± 14.88	ns	ns
*m*-**HBNs (4–6)**	**4**	Me	16.56 ± 3.33	***	ns	118.05 ± 8.19	ns	ns
	**5**	*tert-*Bu	4.15 ± 1.48	***	ns	108.56 ± 7.42	ns	ns
	**6**	Bn	5.56 ± 1.09	***	–	136.17 ± 6.64	ns	–
o-**HBNs (7–9)**	**7**	Me	176.58 ± 15.09	***	***	109.44 ± 4.40	ns	ns
	**8**	*tert-*Bu	58.62 ± 2.86	**	***	106.69 ± 3.62	ns	ns
	**9**	Bn	45.32 ± 7.48	**	**	123.40 ± 7.05	ns	ns
	**PBN**	*–*	81.21 ± 14.39	*–*	***	143.47 ± 9.29	*–*	ns
	**NAC**	*–*	5.16 ± 1.60	***	ns	113.39 ± 5.87	ns	ns

The estimation of EC_50_ (in µM) and maximal activities (in % neuroprotection) values were performed by a weighted nonlinear regression of minimum squares using logistic curves, as is described in the “Statistical Analysis” section of “Neuroprotection Assessment Assays”. Values are the mean ± S.E.M. Data analysis was carried out with SigmaPlot v.12., and ANOVA one-way to get the significant statistics of **HBNs 1–9** respect to **PBN**, or to **HBN6**. Differences are statistically significant when *P* ≤ 0.05. EC_50_ and maximal activities were calculated from the data obtained from three experiments, each one in triplicate. The statistics compares differences with **PBN** or **HBN6** at *P  <  0.05, **P  <  0.01 and ***P < 0.001 (one-way ANOVA, followed by Holm–Sidak analysis as a post hoc test.

As the highest neuroprotective activity (maximal activities) was similar in all cases, we can conclude, by regarding the EC_50_ values, that **HBNs 4–6 **bearing the nitrone motifs in *meta* position gave the best neuroprotection, followed by **HBNs 2–3** bearing the nitrone motifs in *para* position, and **HBNs 7–9** bearing the nitrone motifs in *ortho* position. The high neuroprotection observed for **HBNs 4–6** exceeds that of the parent **PBN** and is very similar to that of *N*-acetyl-*L*-cysteine (**NAC**) (EC_50_ = 5.16 ± 1.60 μM). From the structure–activity relationship (SAR) point of view, note that, among the bis-nitrones of the same group, **HBNs** having a benzyl or *tert-*butyl group at the nitrogen atom of the nitrone motif systematically afforded a higher neuroprotection.

### Neuroprotection analysis in an OGD model

**Figure 3:**
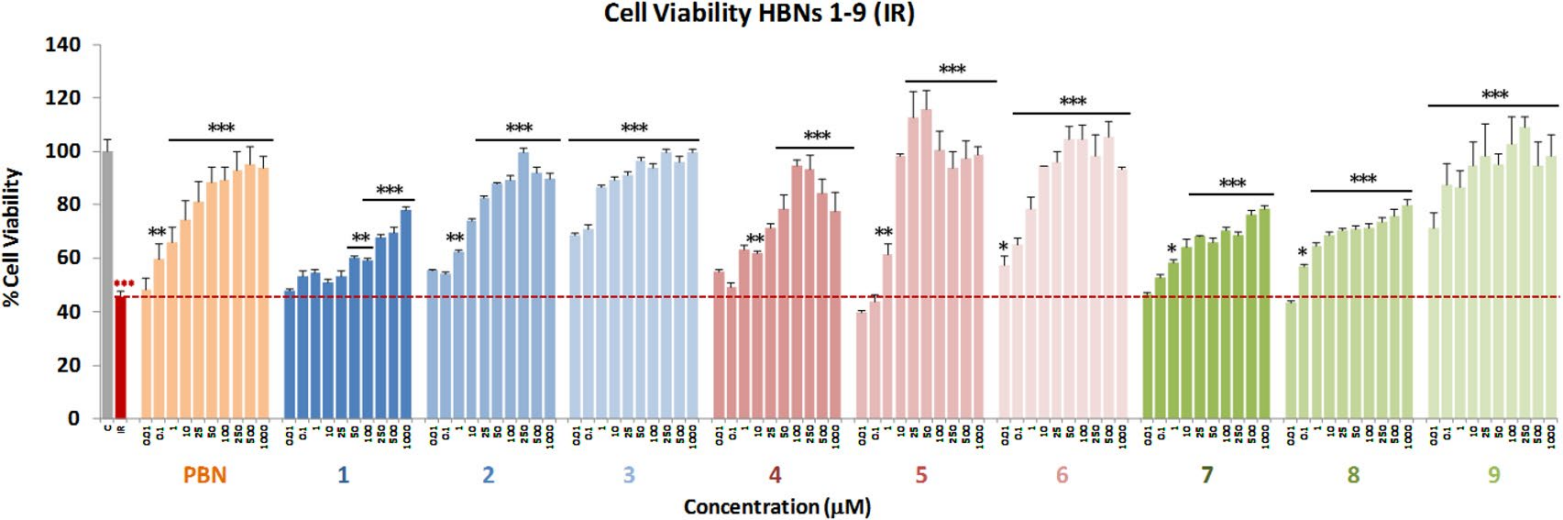
Neuroprotective effect of **HBNs 1–9** on neuroblastoma cells viability after OGD (4 h) and reperfusion (24 h) (IR 24 h). Bars show % cell viability after treatment with IR, with, or without, **HBNs 1–9** and **PBN**, at the indicated concentrations. Values are the mean ± SEM of three experiments, each one performed in triplicate. The statistics compare the effect of IR on its control (red ***) or the effect of the different compounds after IR (24 h) with IR in the absence of these compounds (black ***). Data were statistically analyzed by one-way ANOVA, followed by Holm-Sidak as test post hoc. *P < 0.05; **P < 0.01; ***P < 0.001. (*t*-Bu = *tert*-butyl).

Next, the neuroprotective effect of **HBNs 1–9** was evaluated in an in vitro oxygen glucose deprivation (OGD) model, followed by ischemic reperfusion (IR)^29^. Tested compound concentrations ranged from 0.01 to 1,000 μM, after IR. After OGD (I) (4 h), a loss of metabolic activity between 50–80% was observed, showing a small cell recovery after 24 h reperfusion (IR) of 38% to 61% (49.29 ± 3.26; mean ± SEM; n = 16). **HBNs 1–9** (Fig. 3) were able to partially or even totally reverse the cell loss of metabolic activity induced by IR, in a concentration-dependent manner. These data revealed that **HBN5** and **HBN6** were the most potent bis-nitrones. Among **HBNs 1–3**, **HBNs 2** and **3** provided 60% neuroprotection, regardless of the dose. Furthermore, **HBNs 7–9** afforded a concentration–response curve, with the best neuroprotection reached in the 25–100 μM range. Strikingly, **HBN9** showed high neuroprotection, in the same range than **HBN5** and **HBN6**. To sum up, in the OGD experiment, **HBN5** and **HBN6** showed the best neuroprotective profile, in good agreement with the results observed with the inhibitors of the mitochondrial ETC.

Based on these encouraging results, we have determined the EC_50_ and the highest neuroprotective activities for **HBNs 1–9** comparing them with that of **PBN** and **NAC**. As shown in Table 2, the EC_50_ values, from the lowest to the highest neuroprotective nitrone, follows the order: **HBN3** ≤ **HBN6** ≤ **HBN5** < **HBN9**. However, and based on the observed highest neuroprotective activity (maximal activities), this order was as follows: **HBN3 **< < **HBN6 **≤ **HBN 5** ≤ **HBN 9**. Then, given that **HBN3**, despite having the lowest EC_50_, has a low maximal activity, we could conclude that the neuroprotective capacity of **HBN3** is similar to that of** HBN9**, a compound that shows a high maximal neuroprotective activity at a higher EC_50,_ and that both **HBNs** have lower overall neuroprotective capacity than **HBN6** and **HBN5**, both with a low EC_50_ and a high maximal neuroprotective capacity.

**Table 2:** Neuroprotective effect of **HBNs 1–9**, **PBN** and **NAC** after OGD-IR in human neuroblastoma SH-SY5Y cells.

	HBN	R	Neuroprotection (EC_50_ ± SEM), μM	*P* < (PBN)	*P* < (HBN6)	Maximal Activity (Mean ± SEM), %	*P* < (PBN)	*P* < (HBN6)
*p*-**HBNs (1–3)**	**1**	Me	227.07 ± 15.92	***	***	68.55 ± 3.96	*	***
	**2**	*tert*-Bu	45.09 ± 4.73	ns	***	77.82 ± 2.46	ns	***
	**3**	Bn	0.78 ± 0.09	**	ns	69.01 ± 0.87	*	***
*m*-**HBNs (4–6)**	**4**	Me	38.85 ± 4.05	ns	**	78.21 ± 2.23	ns	***
	**5**	*tert-*Bu	1.70 ± 0.18	**	ns	113.31 ± 4.69	***	ns
	**6**	Bn	1.24 ± 0.23	**	–	104.07 ± 3.06	**	–
o-**HBNs (7–9)**	**7**	Me	20.36 ± 1.21	ns	*	68.28 ± 2.13	**	***
	**8**	*tert-*Bu	20.58 ± 4.66	ns	*	55.43 ± 3.49	***	***
	**9**	Bn	10.14 ± 0.66	*	*	120.41 ± 6.63	***	ns
	**PBN**	–	42.01 ± 5.41	–	**	82.54 ± 6.23	–	**
	**NAC**	–	2.58 ± 0.91	**	ns	110.30 ± 2.81	**	ns

The estimation of EC_50_ (in µM) and maximal activities (in % neuroprotection) values were performed by a weighted nonlinear regression of minimum squares using logistic curves, as is described in the “Statistical Analysis” section of “Neuroprotection Assessment Assays”. Values are the mean ± S.E.M. Data analysis was carried out with SigmaPlot v.12., and ANOVA one-way to get the significant statistics of **HBNs** respect to **PBN**, or to **HBN6**. Differences are statistically significant when *P* ≤ 0.05. EC_50_ and maximal activities were calculated from the data obtained from three experiments, each one in triplicate. The statistics compares differences with **PBN** or **HBN6** at *P < 0.05, **P < 0.01 and ***P < 0.001 (one-way ANOVA, followed by Holm–Sidak analysis as a post hoc test.

From the SAR point of view, note that: (1) the best neuroprotective **HBNs 3**,** 6** and **9** bear a benzyl group at the nitrogen atom of the nitrone motif, (2) **HBN9 **bears the two nitrone motifs in an *ortho* arrangement at the aromatic ring, and (3) the *meta* relative position of nitrones, present in **HBN5** and **HBN6**, is the preferred arrangement to provide an effective neuroprotection. Moreover, the neuroprotection afforded by **HBN5** and **HBN6** is very similar to that of **NAC** (EC_50_ = 2.58 ± 0.91 μM).

### Effect of HBNs on necrotic and apoptotic cell death induced by OGD

**Figure 4:**
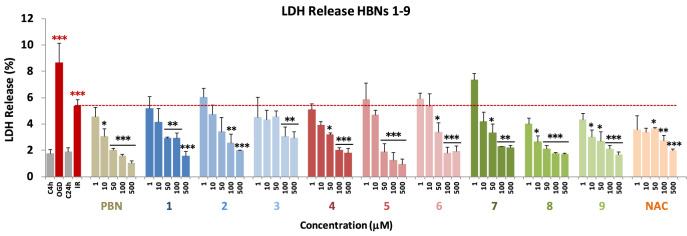
Effect of **HBNs 1–9** on the LDH release in SH-SY5Y cells after IR. Bars show % LDH release after OGD (4 h) and IR (24 h), without treatment (IR 24 h) or treated with **HBNs1–9**, **PBN** and **NAC**, at the indicated concentrations. Values are the mean ± SEM of three experiments, each one performed in triplicate, and compare the effect of OGD and IR on respective controls, C4h and C24h, respectively (red ***) or the effect of the different compounds after IR (24 h) with IR (24 h) in the absence of these compounds (black ***). Data were statistically analyzed by one-way ANOVA, followed by Holm-Sidak as test post hoc. *P < 0.05; **P < 0.01; and ***P < 0.001.

During an ischemic stroke, there is massive cell death due to necrosis, and, as a consequence, the plasma membrane is broken or significantly permeabilized^30^. Under these circumstances, lactate dehydrogenase (LDH), a soluble cytosolic enzyme, easily crosses the damaged membrane, and for this reason, it is possible to determine the extent of the cell necrosis taking place in the OGD experiment by comparing its extracellular to its intracellular activity. As shown in Fig. 4, from the values obtained from the measurement of the LDH release after OGD for 4 h, followed by 24 h reperfusion (IR) on neuroblastoma cells, by adding **HBNs 1–9** at 1–500 µM concentrations (**PBN** and **NAC** as the reference compounds), we concluded that all **HBNs**, with the exception of **HBN3**, **PBN** and **NAC**, significantly decreased the release of LDH, reaching 100% of the LDH activity inhibition (Fig. 4). **HBNs 1–3** were, in general, less potent than **HBNs 4–6**, whereas **HBN8** and **HBN9** were the most efficient bis-nitrones (Fig. 4). Despite that, **HBNs 1–9** exhibited a rather similar inhibitory potency of LDH activity than **PBN** and** NAC**.

**Figure 5:**
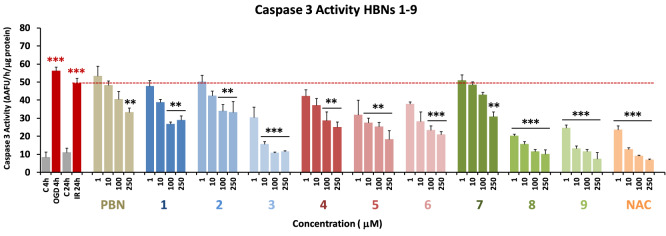
Effect of **HBNs 1–9** on caspase-3 activity in SH-SY5Y neuroblastoma cells after IR. Bars show caspase 3 activity expressed as ΔAFU/μg protein/min after OGD (4 h) and IR (24 h) alone or treated with **HBNs 1–9**, **PBN** and **NAC**, at the indicated concentrations. Values are the mean ± SEM of three experiments, each one performed in triplicate, and compare the effect of OGD and IR on respective controls (C4h and C24h, respectively) (red ***) or the effect of the different compounds after IR treatment with IR alone, in the absence of these compounds (black ***). Data were statistically analyzed by one-way ANOVA, followed by Holm-Sidak as test post hoc. *P < 0.05, **P < 0.01 and ***P < 0.001. UAF = Arbitrary Fluorescent Units.

Next, and in order to evaluate the extent of cell death by apoptosis, we determined the caspase-3 activity, by using DEVD-AMC as a substrate, which affords fluorescent AMC upon hydrolysis. So, after OGD (4 h), and adding **HBNs 1–9**, **PBN** and **NAC**, at 1–250 μM concentration doses, followed by IR (24 h), the cells were lysated, DEVD-AMC was added, and the fluorescence measured. As shown in Fig. 5, it can be concluded that, in general, the tested compounds protect less efficiently from the apoptotic than from necrotic cell death. Among the *para-***HBNs**, **HBN3** was the best agent, as the caspase-3 activity was reduced at 10 μM dose. **HBN5**, **HBN6**, **HBN8** and **HBN9** showed also potent antiapoptotic activity, being *ortho-***HBN8** and **HBN9**, which bear *tert*-Bu and Bn substituents, respectively, more potent than the corresponding *meta*-**HBN5** and **HBN6**. Both, the antiapoptotic and antinecrotic effects of the most potent **HBNs** (**HBN8**, **HBN9**, **HBN5** and **HBN6**), were very similar to those found for **NAC**. However, the antiapoptotic effect of **HBNs 1–9** was greater than that of** PBN**, despite the fact that they have a similar anti-necrotic effect to this base nitrone.

### Basal neurotoxicity of HBNs

**Figure 6:**
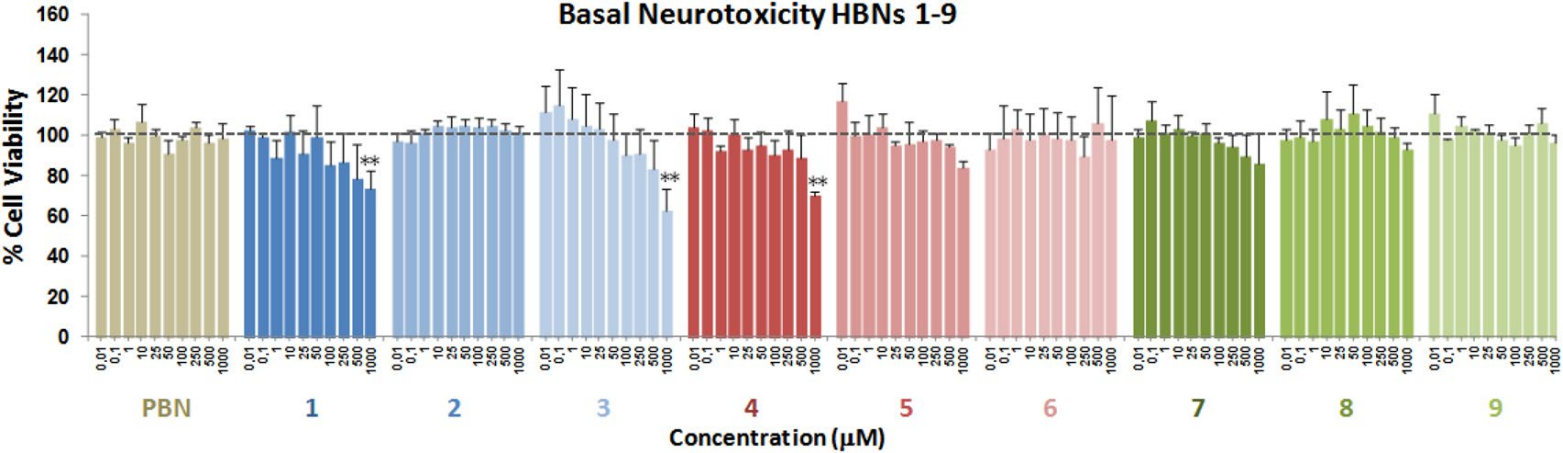
Effect of **HBNs 1–9** and **PBN** on human neuroblastoma SH-SY5Y cell viability under basal conditions. Bars represent % of cell viability in the presence of the ligands at the indicated concentrations. Cell viability for the untreated cells (C) was assigned 100% (100 ± 5.52%; data not shown). Values are the mean ± SEM of five experiments, each one in triplicate. The statistics shows the neurotoxic effects of the ligands against control a **P < 0.01 (one-way ANOVA).

Due to the observed decrease of the neuroprotective effect by **HBNs 4–6** (Fig. 3) or the effect on the LDH release by **HBN3** (Fig. 4) at the highest concentrations tested (250–1,000 μM), the possible neurotoxicity of **HBNs** was investigated. The experiments were carried out by measuring the cell viability with XTT, but without adding any toxic insult. As shown in Fig. 6, none of the **HBNs**, at 250–1,000 μM doses, with the exception of **HBN3**, (62.76 ± 10.57% cell viability at 1 mM; *P* < 0.001 versus 100% C), **HBN4** (70.24 ± 1.73% at 1 mM; *P* < 0.001 test de ANOVA), and **HBN1** (73.65 ± 8.66% at 1,000 μM; *P* < 0.001, test de ANOVA), were neurotoxic.

### Antioxidant capacity of HBNs 1–9: production and scavenging of radical superoxide radical in human neuroblastoma SH-SY5Y cells

**Figure 7:**
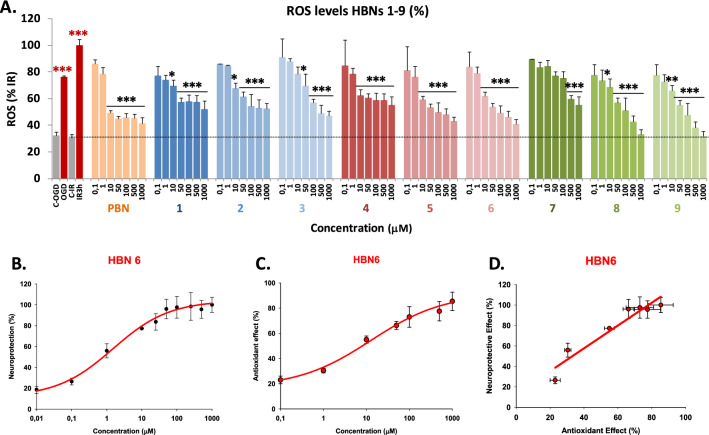
Inhibitory effects of **HBNs 1–9 **and **PBN** on ROS (superoxide) production in SHSY5Y human neuroblastoma cell cultures exposed to OGD (4 h) and 3 h reperfusion (IR). (**A**) Bars show % ROS formed after OGD and IR with, or without, **HBNs 1–9** or **PBN**, at the indicated concentrations. Values are then mean ± SEM of three experiments, each one performed in triplicate. Values for ROS in basal conditions was calculated as 0.42 ± 0.09 UAF/min/150.000 cells (n = 16). The statistics compares the effect of OGD or IR against the corresponding controls (red) or the effect of the different compounds respect to IR (black) at *P < 0.05, **P < 0.01, ***P < 0.001 (one-way ANOVA followed by Holm–Sidak analysis post hoc). ns, no significant. (**B**, **C**) Data analysis of the neuroprotective (**B**) and antioxidant effect (**C**) of **HBN6** (EC_50′s_) on the metabolic cell activity lost (**B**) or the increase in ROS levels (**C**) induced by IR, carried out with SigmaPlot v.12 (see Material and Methods). Values represent the mean ± SEM after three experiments, each one in triplicate. (**D**) Correlation analysis between neuroprotective and antioxidant effects of **HBN6**. Straight line equation, correlation coefficient (r) and statistical significance of regression analyses are indicated in the plot. Regression analysis and statistics were performed by the Pearson Product Moment Correlation Test, carried out with SigmaPlot v.12.

The results shown in the previous sections prompted us to investigate whether the observed neuroprotection was a consequence of their capacity to act as antioxidants and ROS scavengers, particularly of superoxide radical anion (O_2_^·−^). O_2_^·−^ detection was carried out by using dihydroethidium (DHE), after OGD (3 h) and IR (3 h), with or without **HBNs 1–9**, including **PBN** and **NAC** as standards. Compound concentrations from 0.1 to 1,000 μM were tested, after IR. As shown in Fig. 7A, ROS level production after IR (1.46 ± 0.19 UAF/min/150.000 cells; mean ± SEM; n = 16) was higher (*P* < 0.05, one way Anova test) than ROS production after OGD alone (1.14 ± 0.07 UAF/min/150.000 cells mean ± SEM; n = 16). As expected, **HBNs 1–9** were able to partially or totally reverse the increase in ROS levels induced by IR, in a concentration-dependent manner (Fig. 7A). The analyses of concentration–response curves and calculations of EC_50_ and the highest antioxidant activities for **HBNs 1–9** and **PBN** (a graphic example is presented for **HBN6** in Fig. 7C), is shown in Table 3. The EC_50_ values, from the lowest to the highest, follows the order: **NAC** ≤ **HBN 4** ≤ **PBN** ≤ **HBN6** ≤ **HBN 5** < **HBN2** ≤ H**BN1** < < **HBN3** < < **HBN 9** < < **HBN8 **< < < **HBN7**. As the highest neuroprotective activity (maximal activities) was lower for **HBNs 1–4** and higher for**HBNs 5–6** and **HBN8–9**, we conclude that, regarding the antioxidant capacity against IR-induced superoxide production, *m*-**HBNs 4–6** exhibit the best antioxidant properties followed by *p*-**HBNs 1–3**, whose effect is very similar to that of **PBN**. However, the fact that *o*-**HBN8** and *o*-**HBN9** have higher maximal activity than the other **HBNs**, despite their higher EC_50_, makes them highly antioxidant nitrones with a similar activity to those of **HBNs 1–3**, which, despite their lower EC_50_, exhibit the lowest maximal activity.

**Table 3:** Antioxidant effect of **HBNs 1–9**, **PBN **and **NAC** after OGD-IR in human neuroblastoma SH-SY5Y cells.

	HBN	R	Antioxidant effect (EC_50_ ± SEM), µM	*P* < (PBN)	*P* < (HBN6)	Maximal Activity (Mean ± SEM), %	*P* < (PBN)	*P* < (HBN6)
*p*-**HBNs (1–3)**	**1**	Me	13.41 ± 3.49	ns	ns	69.10 ± 3.25	*	***
	**2**	*tert*-Bu	9.06 ± 0.65	ns	ns	69.10 ± 3.25	*	***
	**3**	Bn	38.52 ± 5.25	ns	ns	81.43 ± 2.99	ns	**
*m*-**HBNs (4–6)**	**4**	Me	3.31 ± 0.99	ns	ns	61.38 ± 1.85	**	***
	**5**	*tert-*Bu	6.73 ± 0.57	ns	ns	93.48 ± 4.47	*	ns
	**6**	Bn	5.91 ± 1.09	ns	–	95.78 ± 3.63	**	–
o-**HBNs (7–9)**	**7**	Me	248.59 ± 61.03	***	***	81.64 ± 4.45	ns	**
	**8**	*tert-*Bu	108.97 ± 9.88	**	**	106.27 ± 7.19	***	ns
	**9**	Bn	68.68 ± 16.12	*	*	107.53 ± 4.27	***	*
	**PBN**	–	3.66 ± 0,57	–	ns	80.56 ± 1.48	–	**
	**NAC**	–	3.23 ± 0.35	ns	ns	112.35 ± 3.32	**	*

The estimation of EC_50_ (in µM) and maximal activities (in % neuroprotection) values were performed by a weighted nonlinear regression of minimum squares using logistic curves, as is described in the “Statistical Analysis” section of “Neuroprotection Assays”. Values are the mean ± S.E.M. Data analysis was carried out with SigmaPlot v.12., and ANOVA one-way to get the significant statistics of **HBNs 1–9** respect to **PBN**, or to **HBN6**. Differences are statistically significant when *P* ≤ 0.05. EC_50_ and Maximal Activities were calculated from the data obtained from three experiments, each one in triplicate. The statistics compares differences with **PBN** or **HBN6** at *P < 0.05, **P < 0.01 and ***P < 0.001 (one-way ANOVA, followed by Holm–Sidak analysis as a post hoc test.

In summary, and from the SAR point of view, once again **HBN5 **and **HBN6**, bearing *tert*-Bu and Bn, substituents, respectively, were confirmed to be the most potent bis-nitrones of the entire series. Finally, although the antioxidant effects of **HBN5** and **HBN6** were very similar to that of **PBN**, the fact that **PBN** has a lower maximum antioxidant activity, led us to conclude that both nitrones exceed **PBN** as ROS scavengers.

Finally, to examine whether the antioxidant effect of **HBNs** could be responsible for their neuroprotective effect, we performed a linear correlation analysis of neuroprotective power (Fig. 7B) versus the antioxidant capacity (Fig. 7C), as shown in Fig. 7D for **HBN6**. In all cases, there was a very significant correlation between both effects, with Pearson correlation coefficients (r) ranging from 0.807 to 0.983, and with a statistical significance > 0.001 in the case of nitrones with *tert*-Bu and Bn substituents and < 0.01 or < 0.05 for **HBNs** with Me substituent and **PBN** (data not shown).

To sum up, it becomes clear that the results of the ROS trapping experiment are in good agreement with the neuroprotection analyses and firmly confirm that **HBN5** and **HBN6** are the best and most balanced bis-nitrones of the studied series in terms of neuroprotection (OGD plus IR, and O/R) and antioxidant power. In addition, the antioxidant power of **HBN5** and **HBN6** is very similar to the antioxidant power of **NAC** (EC_50_ = 3.23 ± 0.35 μM).

Based on the neuroprotection results, we have also investigated the antioxidant power analysis of **HBNs 5–9** on diverse antioxidant tests, using **PBN**, **NDGA** and **Trolox** as standards for comparative purposes.

### Antioxidant tests

As shown in Table 4, **HBN9** was able to inhibit 80% lipid peroxidation (LP), in the same range as **Trolox** (88%), in the same experiment, as well as lipoxygenase (LOX) (85 µM) and 2,2′-azino-bis(3-ethylbenzthiazoline-6-sulfonic acid (ABTS^+.^) (23%), albeit in a poorer extent than **NDGA** (0.45 µM), and **Trolox** (91%), respectively. Note also that **HBN6** was the most potent hydroxyl radical scavenger (81%), overcoming **HBN5** and **HBN9**, and in the same range that **Trolox** (83%). Finally, compared to **PBN**, **HBN5**, **HBN6** and **HBN9** showed more potent balanced antioxidant capacity, in good agreement with the higher calculated ClogP values, which are also consistent with the neuroprotection results (see above).

**Table 4:** Antioxidant activity of **HBNs 5–9**, **PBN**, **Trolox **and **NDGA**.

HBNs/standards	ClogP^a^	ILPO (%)	LOX inhibition (IC_50_ [μM]/%)	Scav. activity for ^·^OH (%)	ABTS^+.^ (%)
**PBN**	3.02	11	23	no	5
**HBN5**	**4.51**	**55**	6	**67**	no
**HBN6**	**4.96**	**37**	**29**	**81**	no
**HBN7**	2.56	64	60 µM	29	22
**HBN8**	0.61	46	57.5 µM	59	4
**HBN9**	**4.95**	**80**	85 µM	**16**	**23**
**NDGA**	nd	nd	0.45 µM	nd	nd
**Trolox**	nd	88	nd	83	91

Bold is for emphasis

Nitrones tested at 100 µM; Values are means of three or four different determinations. No, no activity under the experimental conditions. Means within each column differ significantly (*P* < 0.05).

*nd* not determined, *no* no activity.

^a^Biobyte BioByte Corporation, C-QSAR database, 201 W Fourth Str., Suite # 204, Claremont CA 91711–4707, USA.

### Contribution of HBN6 to brain damage prevention

**Figure 8:**
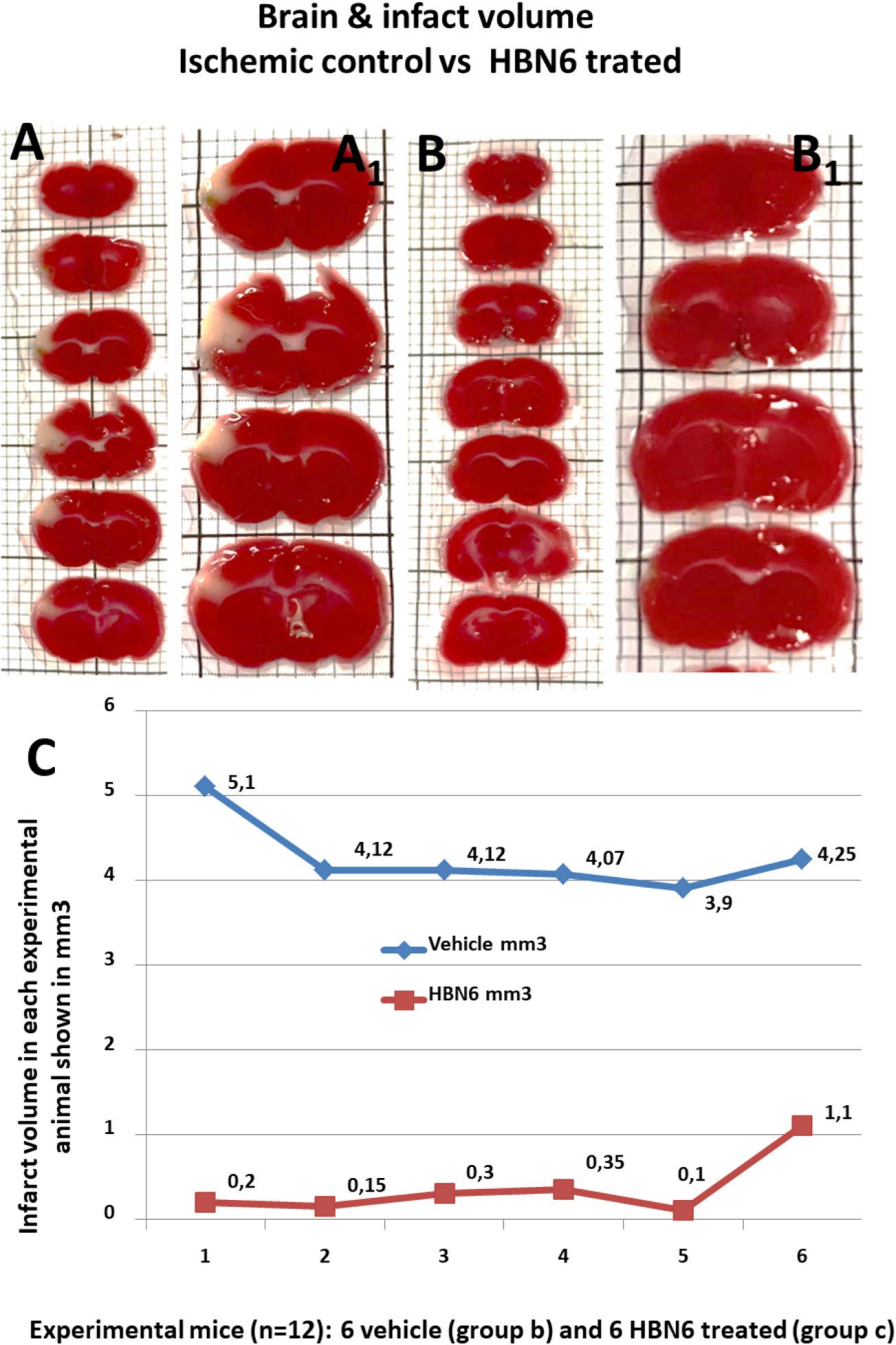
Treatment with **HBN6** reduces the infarct volume after pMCAO, frontal branch, in mice. Mice were subjected to pMCAO, and volumes of both the whole brain and the infarcted region were measured 48 h after ischemic onset, by TTC-stained of serial 1 mm-thick coronal sections. Representative stacks of TTC-stained sections are shown for each experimental group: b (**A**, **A1**_,_ vehicle treated) and c (**B**, **B1**_,_ **HBN6** treated), see point 5.3 in the experimental part. (**A1**) and (**B1**) are high-power magnifications of same slices shown in A and B, respectively. No differences in total brain volumes were detected between group´s b and c. In striking contrast, mice of the group b (**A**, **A1**; saline buffer containing 29% dimethyl sulfoxide, DMSO) reveal a larger unstained area of ischemic tissue in the neocortex when compared to their **HBN6** treated littermates of the group c (**B**, **B1**; 100 mg/kg HBN6 dissolved in the same vehicle volume as shown in group b) that lack detectable cortical infarct. (C) Data of six animals (n = 6) for each group, vehicle (group b) and **HBN6** (group c) intraperitoneally administered. Groups were compared by a two-tailed Student’s t-test (*P* < 0.001).

Permanent ischemia models reflect the most frequent variants of stroke in patients who are outside of therapeutic windows, or are non-responders to recombinant tissue plasminogen activator, or surgical thrombectomy. In addition, permanent ischemia (no reperfusion) has also been associated with substantial accumulation of ROS^31^. Permanent middle cerebral artery occlusion (pMCAO) is a commonly used stroke model in mice^32^. Using the pMCAO procedure^33^, we analyzed the in vivo contribution of **HBN6 **to brain damage prevention. As expected, animals in the sham operated group showed no infarct (not shown). In striking contrast, groups subjected to pMCAO showed, 48 h after the occlusion procedure, infarcted regions which included exclusively the cerebral cortex (Fig. 8). The average size of the infarcted brain area was of 4.26 mm^3^ ± 0.2 mm^3^ (mean ± S.E.M.; n = 6) in vehicle treated animals, a value significantly greater than that for the **HBN6** treated group, 0.37 ± 0.15 mm^3^ (media ± SEM) (n = 6; *P* < 0.001 two-tailed Student’s t test assuming equal variances) (Fig. 8A,B). Considering that the total brain volume for the vehicle-treated mice was 458.42 ± 18.41 mm^3^ and for the mice treated with **HBN6** 458.26 ± 26.16 mm^3^ (mean ± SEM; n = 6; ns, two-tailed Student's t-test), this area represents 0.92 ± 0.05% and 0.088 ± 0.03% of the total brain volume, for vehicle and **HBN6**-treated animals, respectively (n = 6; *P* < 0.001 two-tailed Student’s t test).

### Computational studies

**Figure 9:**
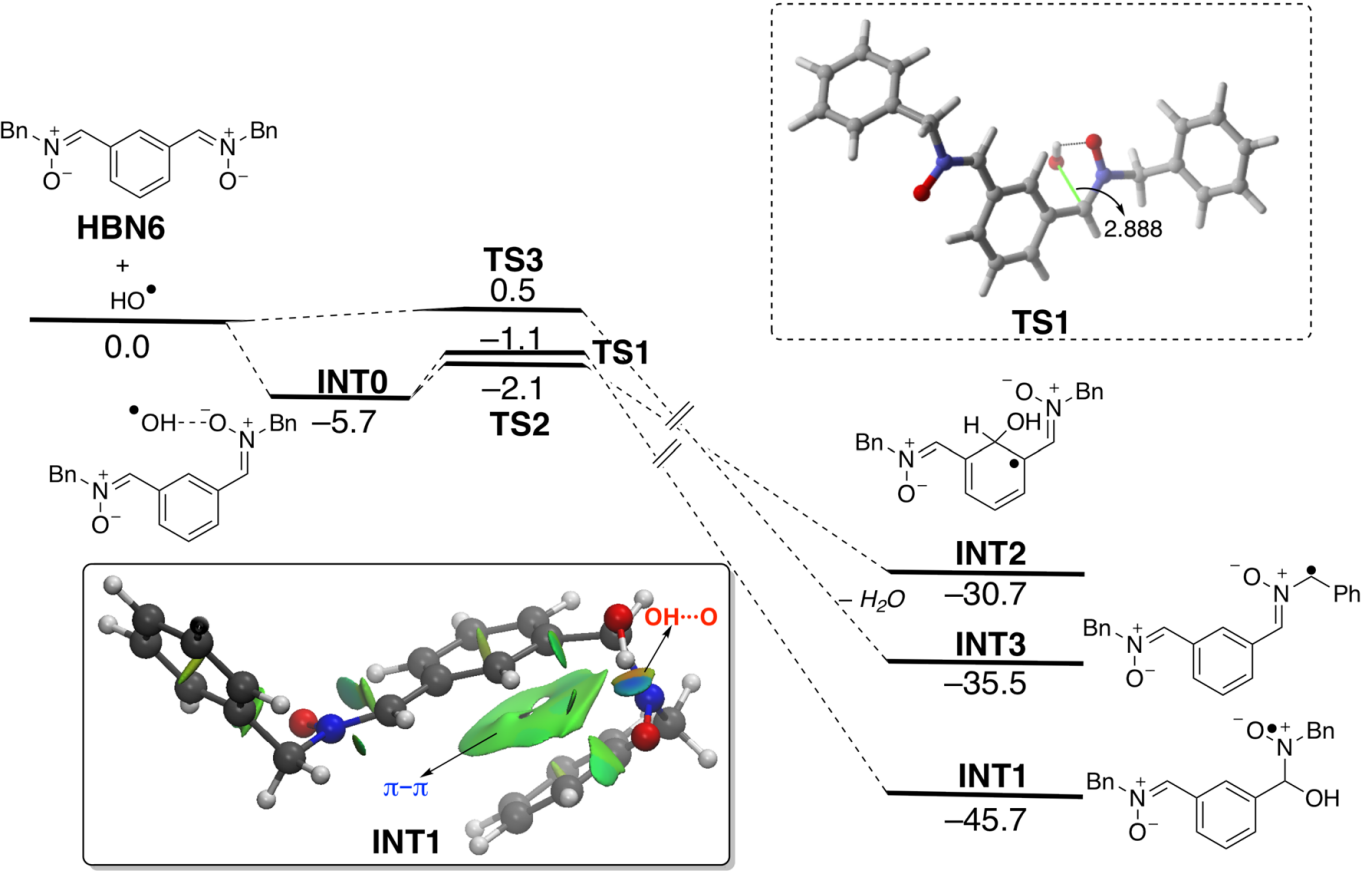
Computed reaction profile for the reaction of bis-nitrone **HBN6** and HO^·^. Relative free energies (∆G, at 298 K) and bond distances are given in kcal/mol and angstroms, respectively. Inset: NCI plot computed for radical intermediate INT1. All data have been computed at the B3LYP-D3/def2-SVP level.

Density Functional Theory (DFT) calculations were carried out at the dispersion corrected B3LYP-D3/def2-SVP level (see computational details in the Supplementary Information) to gain more insight into the higher neuroprotective response of **HBN6** as compared to the parent nitrone **PBN**. To this end, we first explored the reaction between the oxygen-centered radical HO^·^ and **HBN6**. Three different pathways were envisaged (see Fig. 9), namely the addition of the radical to the carbon atom of the nitrone moiety leading to INT1, the addition to the aryl carbon atom placed in adjacent position to both nitrone moieties (leading to INT2), and the hydrogen abstraction reaction leading to INT3 which releases a water molecule.

From the data in Fig. 9, it becomes evident that the processes are thermodynamically controlled in view of the rather low activation barriers computed for the different possible pathways. Interestingly, the thermodynamically preferred pathway involves the addition of the hydroxyl radical to the electrophilic C=N bond of the nitrone therefore leading to the radical intermediate INT1. The higher stability of this species with respect to INT2 or INT3 may be in part ascribed to the occurrence of a stabilizing π–π non-covalent interaction involving the phenyl group of the benzyl group and the central aryl group, as easily visualized by means of the corresponding NCI plot (see green surface in the inset of Fig. 9).

Table 5 gathers the computed reaction energies of the different pathways involving **HBN6** and **PBN**. Data for **HBN5** are also included to enable a direct comparison (i.e. **PBN** and **HBN5** possess a *tert-*Bu group as a nitrone substituent). As clearly seen from the computed energy values, all the possible pathways are less favored for the processes involving the parent **PBN**, which is fully consistent with the lower neuroprotective response observed for this nitrone. Except for the hydrogen abstraction reaction, the addition of OH· is only slightly favored for **HBN6** as compared to its *t-*Bu substituted counterpart **HBN5**, which again is also consistent with the slightly higher response of the **HBN6** as compared to **HBN5** (see above). Therefore, it can be concluded that the neuroprotective ability of the considered bis-nitrones may be directly related to the exergonicity of their initial reaction (mainly addition reaction) with the corresponding ROS.

**Table 5:** Computed free reaction energies (∆G_R_, at 298 K, in kcal/mol) for the reactions involving **HBN6**, **PBN** and **HBN5** and radicals HO^·^. All data have been computed at the B3LYP-D3/def2-SVP level.

Reaction path	HBN6	PBN	HBN5
INT1	− 45.7	− 43.6	− 45.1
INT2	− 30.7	− 22.7	− 30.4
INT3 + H_2_O	− 35.5	− 13.1	− 14.0

### Virtual ADME analysis

Drug-like properties were determined for** HBNs 1–9** and **PBN** as reference compound. We have used the QikProp software [QikProp, version 5.1, Schrodinger, LLC, New York, NY, 2017-1], and results are summarized in Table 1S (Supplementary Information). Out of ten compounds, seven nitrones were found to have no Lipinski’s rule^34^ violation and three nitrones showed one violation (Table 1S). According to Lipinski’s rule^34^, the partition coefficient (QPlogPo/w) value should be ≤ 5. For nitrones bearing the *N*-benzyl moiety (**HBN3**, **HBN6** and **HBN9**) QPlogPo/w values range from 6.048 to 6.247. The number of hydrogen bond donors (NdonorHB) and hydrogen bond acceptors (NaccptHB) for all the nitrones were in agreement with the drug-likeness requirements of the Lipinski’s rule of five^34^ (NdonorHB ≤ 5, NaccptHB ≤ 10). The predicted central nervous system (CNS) activity with a score range from − 2 (inactive) to + 2 (active) indicated that bis-nitrones **HBNs 1–9** had lower activity in the CNS (predicted value = 0) than** PBN** (predicted value = 1). Molecular volume is another crucial factor for binding at the active site. It was found that all bis-nitrones have molecular volumes between 667.298 and 1,188.993 Å^3^ (the reference value of molecular volume is 500–2000 Å^3^). The aqueous solubility (QPlogS) of a compound significantly affects its absorption and distribution characteristics. Typically, a low solubility goes along with a bad absorption. Only **HBN3** (QPlogS: − 6.81) and **HBN6 **(QPlogS: − 6.87) presented solubility values out of the limits (− 6.5 to 0.5). This is the reason why these two nitrones showed one violation of the rule-of-three (ROT)^35,36^. QPPCaco, which predicts permeability of a molecule for the gut-blood barrier through passive transport, is also one important factor to be considered. Compounds with high Caco-2 permeability (QPPCaco) are easy to absorb. Caco-2 cell permeability prediction of the tested bis-nitrones indicated excellent results, predicting good intestinal absorption. The prediction of Blood Brain Barrier (BBB) permeability, determined by the logBB was also assessed. Compounds with logBB below − 1 are poorly distributed to the brain and are improbable to operate as effective CNS drugs. All bis-nitrones displayed logBB > − 1, pointing towards potential BBB permeability. The number of likely metabolic reactions (metab) is necessary for determining the level of accessibility of compounds to their target sites after entering into the blood stream. The predicted average number of possible metabolic reactions indicated that all bis-nitrones possessed metab values in the recommended range (1–8). All bis-nitrones also exhibited an excellent predicted percentage of oral absorption, 100%. The optimum value polar surface area (7–200 Å) holds a great importance on the oral bioavailability of the molecules; in the present study, bis-nitrones were exhibited 18.99–45.83 Å value of PSA, indicating good bioavailability by oral route.

In particular, for **HBN6** and **PBN**, the more significant observed data were the following: (1) Total Solvent Accessible Surface Area (SASA), in square Å, using a probe with a 1.4 Å radius: 686.317 and 429.378, respectively (limits 300.0–1,000.0); (2) Estimated number of hydrogen bonds (donorHB) that would be accepted by the solute: 0 for both compounds (limits: 0.0–6.0); (3) Predicted octanol/water partition coefficient (QPlogPo/w): 6.244 and 3.450, respectively (limits − 2.0 to 6.5); (4) Predicted aqueous solubility. S, in mol/dm^3^, is the concentration of the solute’s saturated solution that is in equilibrium with crystalline solid (QPlogS): − 6.873 and − 3.342, respectively (limits − 6.5 to 0.5); (5) Predicted brain/blood partition coefficient (QPlog BB): − 0.601 and 0.197, respectively (limits − 3.0 to 1.2); (6) Van der Waals surface area of polar nitrogen and oxygen atoms (PSA): 43.368 and 18.991, respectively (limits 7.0–200.0); (7) Number of violations of Lipinski's Rule Of Five (ROF): 1 and 0, respectively; number of violations of Jorgensen's rule of three (ROT): 1 and 0, respectively. To sum up, the predicted results indicate that the ADME properties for **HBN6** are quite similar to those of** PBN**, except that **HBN6** must be less soluble in water due to its highest lipophilicity (QPlogPo/w > 5). Nevertheless, an orally active drug should have no more than one violation of the Lipinski's Rule, as it is the case of agent **HBN6**, and consequently, no problems with its bioavailability should be observed.

## Conclusions

In this work we have described the design, synthesis and biological evaluation of nine bis-nitrones **HNBs 1–9** derived from **PBN** for the potential treatment of stroke. The biological evaluation of the bis-nitrones included neuroprotection against Oligomycin A/Rotenone, and in an ischemia in vitro model under OGD conditions in human neuroblastoma SH-SY5Y cells, and diverse antioxidant tests. Our design, supported on literature precedents, was based on the hypothesis that two nitrone motifs installed in the same scaffold should afford higher neuroprotective power than only one nitrone group. For our approach, we have used **PBN **(Fig. 1) as the nitrone standard to implement this strategy. The *homo-bis-nitrones* **HNBs 1–9** are the result of the incorporation a second identical nitrone at *para,* *meta* and *ortho* positions leading to **HBNs 1–3**, **HBNs 4–6** and **HBNs 7–9**, respectively, and bearing methyl, *tert*-butyl or benzyl substituents, as the *N*-alkyl groups at the nitrone motif (Fig. 1). The desired ligands were easily obtained by simple methods from readily available starting precursors. From all the neuroprotection results, we were able to identify bis-nitrone (1*Z*,1′*Z*)-1,1′-(1,3-phenylene)bis(*N*-benzylmethanimine oxide) (**HBN6**), bearing the two nitrone motifs in *meta* position, and two *N*-benzyl groups at the nitrone scaffold, as a potent neuroprotective agent (EC_50_ = 1.24 ± 0.39 µM) with strong hydroxyl radical scavenger power (81%), in the same range as **Trolox** (83%), and a strong capacity to decrease superoxide production in human neuroblastomas cell cultures, which exceeds the neuroprotective and antioxidant capacities of the parent **PBN**. This may be related to the exergonicity of the addition of the oxygen-centered radical to the carbon atom of the nitrone moiety. In silico results allowed us to conclude that **HBN6 **is predicted to be a potential drug candidate, via oral administration, due to its relevant drug-likeness profile, bioavailability, excellent liposolubility and adequate pharmacokinetics, including CNS permeability, although with low water solubility. To sum up, all these observations confirmed that our initial design hypothesis was correct (“*two* *better* *than* *one”*), and could be used as a guideline to design new and more efficient neuroprotective agents for the therapy of stroke.

## Methods

### Chemistry

Compound purification was performed by column chromatography with Merck Silica Gel (40–63 µm) or by flash chromatography (Biotage Isolera One equipment) and the adequate eluyent for each case. Reaction course was monitored by thin layer cromatography (t.l.c.), revealing with UV light (λ = 254 nm) and ethanolic solution of vanillin or ninhydrin. Melting points were determined using a Reichert Thermo Galen Kofler block and are uncorrected. Samples were dissolved in CDCl_3_ or DMSO-*d*_6_ using TMS as internal standard for ^1^H NMR spectra. In ^13^C NMR spectra, CDCl_3_ central signal (77.0 ppm) and DMSO-*d* (39.5 ppm) were used as references. ^1^H-NMR and ^13^C-NMR spectra were obtained in Bruker Avance 300 (300 MHz) and Bruker Avance 400 III HD (400 Hz) spectrometers. Chemical shifts (δ) are given in ppm. Coupling constants (*J*) are given in Hz. Signal multiplicity is abbreviated as: singlet (s), doublet (d), triplet (t), quartet (c), doublet of doublets (dd), triplet of doublets (td), or multiplet (m). IR spectra were recorded on a Perkin-Elmer Spectrum One B spectrometer. Units are cm^−1^. Low resolution mass spectra were recorded on an Agilent HP 1,100 LC/MS Spectrometer, whereas High Resolution mass spectrometry (Exact Mass) was performed in an AGILENT 6,520 Accurate-Mass QTOF LC/MS Spectrometer. Elemental analyses were performed in an Elementary Chemical Analyzer LECO CHNS-932.

### General methods for the synthesis of nitrones

Method A: To a suspension of the bis(tris)carbaldehyde (1 mmol) in dry EtOH (20 mL), anhydrous NaHCO_3_ (3 equiv), Na_2_SO_4_ (4 equiv) and the corresponding *N*-alkylhydroxylamine hydrochloride (3 equiv) were added. The mixture was irradiated at 90 °C, and 15 bar, for the time indicated in each case. Then, the mixture was cooled, the solvent removed, and the crude purified by column chromatography. Method B: As in Method A, but at room temperature (rt). Method C: As in Method A, but in dry THF as solvent.

#### (1*Z*,1′*Z*)-1,1′-(1,4-Phenylene)bis(*N*-methylmethanimine oxide) (**HBN1**)^21^

Following the general Method A, the reaction of terephthalaldehyde (134 mg, 1 mmol) with NaHCO_3_ (252 mg, 3 mmol), Na_2_SO_4_ (568 mg, 4 mmol) and *N*-methylhydroxylamine hydrochloride (250,6 mg, 3 mmol), in EtOH (20 mL), for 1 h, after work-up and purification by column chromatography eluting with MeOH:CH_2_Cl_2_ at 3%, gave **HBN1** (146,6 mg, 76%): mp > 220 °C; IR (KBr) ν 3,423, 1591, 1,418, 1,129 cm^-1^; ^1^H NMR (500 MHz, DMSO-d_6_) δ 8.22 (s, 4 H, H-2, H-3, H-5, H-6), 7.85 (s, 2 H, H-7, 9), 3.77 (s, 6 H, CH_3_); ^13^C NMR (126 MHz, DMSO-d_6_) δ 133.9 (2 C, C-7, C-9), 132.3 (2 C, C-1, C-4), 127.9 (4 C, C-2, C-3, C-5, C-6), 54.6 (2 C, CH_3_); MS (ESI) *m/z*: 193 [M + 1]^+^, 215 [M + Na]^+^, 404 [2 M + Na]^+^. Anal. Calcd for C_10_H_12_N_2_O_2_: C, 62.49; H, 6.29; N, 14.57. Found: 62.48; H, 6.28; N, 14.57.

#### (1*Z*,1′*Z*)-1,1′-(1,4-Phenylene)bis(*N-tert*-butylmethanimine oxide) (**HBN2**)^22^

Following the general Method A, the reaction of terephthalaldehyde (134 mg, 1 mmol) with NaHCO_3_ (252 mg, 3 mmol), Na_2_SO_4_ (568 mg, 4 mmol) and *N*-*tert*-butylhydroxylamine hydrochloride (375 mg, 3 mmol), in EtOH (20 mL), for 3,5 h, after work-up and purification by column chromatography eluting with AcOEt: hexane (2:3), gave **HBN2** (83,4 mg, 30%): mp > 220 °C; IR (KBr) ν 3,434, 1569, 1,361, 1,125 cm^−1^; ^1^H NMR (500 MHz, DMSO-*d*_6_) δ 8.34 (s, 4 H, H-2, H-3, H-5, H-6), 7.86 (s, 2 H, H-7, H-9), 3.31 [s, 18 H, C(CH_3_)_3_]; ^13^C NMR (126 MHz, DMSO-*d*_6_) δ 132.6 (2 C, C-1, C-4), 128.9 (2C, C-7, C-9), 128.3 (4 C, C-2, C-3, C-5, C-6), 71.0 (2 C, C-8, C-10), 28.3 (6 C, C(*C*H_3_)_3_]; MS (ESI) *m/z*: 277 [M + 1]^+^, 299 [M + Na]^+^. Anal. Calcd for C_16_H_24_N_2_O_2_: C, 69.53; H, 8.75; N, 10.14. Found: C, 69.31; H, 8.69; N, 10.13.

#### (1*Z*,1′*Z*)-1,1′-(1,4-Phenylene)bis(*N*-benzylmethanimine oxide) (**HBN3**)^23^

Following the general Method A, the reaction of terephthalaldehyde (134 mg, 1 mmol) with NaHCO_3_ (252 mg, 3 mmol), Na_2_SO_4_ (568 mg, 4 mmol) and *N*-benzylhydroxylamine hydrochloride (477 mg, 3 mmol), in EtOH (20 mL), for 2,5 h, after work-up and purification by column chromatography eluting with AcOEt, gave **HBN3 **(110 mg, 32%): mp > 220 °C; IR (KBr) ν 3,435, 1,570, 1,459, 1,152 cm^−1^; ^1^H NMR (400 MHz, DMSO-d_6_) δ 8.24 (s, 4 H, H-2, H-3, H-5, H-6), 8.12 (s, 2 H, H-7, H-9), 7.54–7.31 (m, 12 H, C_6_H_5_), 5.08 (s, 4 H, H-8, H-10); ^13^C NMR (101 MHz, DMSO-*d*_6_) δ 135.1 (C_6_H_5_), 133.4 (2 C, C-7, C-9), 132.3 (2 C, C-1, C-4), 129.4, 128.9, 128.8 (C_6_H_5_), 128.2 (4 C, C-2, C-3, C-5, C-6), 70.6 (C-8, C-10); MS (ES) *m/z* (%): 350 [M + 1]^+^, 372 [M + Na]^+^. Anal. Calcd for C_22_H_20_N_2_O_2_ 2/7 H_2_O: C, 75.59; H, 5.93; N, 8.01. Found: C, 75.68; H, 6.22; N, 7.88.

#### (1*Z*,1′*Z*)-1,1′-(1,3-Phenylene)bis(*N*-methylmethanimine oxide) (**HBN4**)^24,25^

Following the general Method A, the reaction of isophthalaldehyde (134 mg, 1 mmol) with NaHCO_3_ (252 mg, 3 mmol), Na_2_SO_4_ (568 mg, 4 mmol) and *N*-methylhydroxylamine hydrochloride (250,6 mg, 3 mmol), in EtOH (20 mL), for 1 h, after work-up and purification by column chromatography eluting with MeOH:CH_2_Cl_2_ al 4%, gave **HBN4 **(185,9 mg, 97%): mp 148–150 °C; IR (KBr) ν 3,419, 1584, 1,415, 1,172, 1,157 cm^−1^; ^1^H NMR (400 MHz, DMSO-d_6_) δ 8.92 (s, 1 H, H-6), 8.34 (dd, *J* = 7.9, 1.7 Hz, 2 H, H-4, H-2), 7.90 (s, 2 H, H-9, H-7), 7.49 (t, *J* = 7.9 Hz, 1 H, H-3), 3.80 (s, 6 H, H-8, H-10); ^13^C NMR (101 MHz, DMSO-d_6_) δ 134.0 (2 C, C-7, C-9), 131.6 (2 C, C-1, C-5), 129.1 (2 C, C-4, C-2), 128.9 (C-3), 127.6 (C-6), 54.6 (2 C, C-8); MS (ESI) *m/z*: 193 [M + 1]^+^, 215 [M + Na]^+^, 404 [2 M + Na]^+^. Anal. Calcd for C_10_H_12_N_2_O_2_: C, 62.49; H, 6.29; N, 14.57. Found: 62.21; H, 6.23; N, 14.58.

#### (1*Z*,1′*Z*)-1,1′-(1,3-Phenylene)bis(*N*-*tert-*butylmethanimine oxide) (**HBN5**)^26^

Following the general Method A, the reaction of isophthalaldehyde (134 mg, 1 mmol) with NaHCO_3_ (252 mg, 3 mmol), Na_2_SO_4_ (568 mg, 4 mmol), and *N*-*tert*-butylhydroxylamine hydrochloride (375 mg, 3 mmol), in EtOH (20 mL), for 3,5 h, after work-up and purification by column chromatography eluting with AcOEt: hexane (3:2), gave **HBN5** (248 mg, 90%): mp 147–9 °C; IR (KBr) ν 3,435, 2,976, 1573, 1,361, 1,180 cm^−1^; ^1^H NMR (500 MHz, DMSO-*d*_6_) δ 9.22 (s, 1 H, H-2), 8.39 (dd, *J* = 7.9, 1.7 Hz, 2 H, H-4, H-6), 7.82 (s, 2 H, H-7, H-9), 7.44 (t, *J* = 7.9 Hz, 1 H, H-5), 1.50 [s, 18 H, C(CH_3_)_3_]; ^13^C NMR (126 MHz, DMSO-*d*_6_) δ 131.88 (2C, 1, 3), 129.59 (2C, 4, 6), 128.92 (3C, C-2, 7, 9), 128.50 (C-5), 70.95 (2C, 8, 10), 28.28 (6C, C(CH_3_)_3_]; MS (ESI) *m/z*: 277 [M + 1]^+^, 299 [M + Na]^+^. Anal. Calcd for C_16_H_24_N_2_O_2_: C, 69.53; H, 8.75; N, 10.14. Encontrado: C, 69.26; H, 8.75; N, 9.87.

#### (1*Z*,1′*Z*)-1,1′-(1,3-Phenylene)bis(*N*-benzylmethanimine oxide) (**HBN6**)

Following the general Method A, the reaction of isophthalaldehyde (134 mg, 1 mmol) with NaHCO_3_ (252 mg, 3 mmol), Na_2_SO_4_ (568 mg, 4 mmol) and *N*-benzylhydroxylamine hyrochloride (477 mg, 3 mmol), in EtOH (20 mL), for 2.5 h, after work-up and purification by column chromatography eluting with AcOEt: hexane (3:2), gave **HBN6** (165 mg, 48%): mp 185–187 °C; IR (KBr) ν 3,435,1582, 1,458, 1,175 cm^−1^; ^1^H NMR (500 MHz, DMSO-*d*_6_) δ 8.98 (s, 1H, H-2), 8.29 (dd, *J* = 7.9, 1.7 Hz, 2 H, H4, H-6), 8.12 (s, 2 H, H-7, H-9), 7.51–7.32 (m, 11 H, H-5, C_6_H_5_), 5.06 (s, 4 H, H-8, H-10); ^13^C NMR (126 MHz, DMSO-*d*_6_) δ 135.1 (C_6_H_5_), 133.4 (2 C, C-7, C-9), 131.5 (2 C, C-1, C-3), 129.4 (2 C, C-4, C-6), 129.4 (C-5), 129.0, 128.8, 128.7 (C_6_H_5_), 128.0 (C-2), 70.5 (C-8, C-10); MS (ES) *m/z* (%): 350 [M + 1]^+^, 372 [M + Na]^+^. Anal. Calcd for C_22_H_20_N_2_O_2_: C, 76.72; H, 5.85; N, 8.13. Found: C, 76.59; H, 6.02; N, 8.28.

#### (1*Z*,1′*Z*)-1,1′-(1,2-Phenylene)bis(*N*-methylmethanimine oxide) (**HBN7**)

Following the general Method B, the reaction of phthalaldehyde (134 mg, 1 mmol) with NaHCO_3_ (252 mg, 3 mmol), Na_2_SO_4_ (568 mg, 4 mmol) and *N*-methylhydroxylamine hydrochloride (250,6 mg, 3 mmol), in EtOH (20 mL), for 3 d, after work-up and purification by column chromatography eluting with MeOH:CH_2_Cl_2_ al 6%, gave **HBN7** (140 mg, 73%): mp 153–155 °C; IR (KBr) ν 3,413, 1592, 1,415, 1,170 cm^−1^; ^1^H NMR (400 MHz, DMSO-d_6_) δ 8.99 (dd, *J* = 6.0, 3.5 Hz, 2 H, H-3, H-6), 8.02 (s, 2 H, H-7, H-9), 7.44 (dd, *J* = 6.0, 3.5 Hz, 2 H, H-4, H-5), 3.84 (s, 6 H, H-8, H-10); ^13^C NMR (101 MHz, DMSO-d_6_) δ 130.6 (2 C, C-7, C-9), 129.5 (2 C, C-4, C-5), 128.8 (2 C, C-1, C-2), 127.4 (2 C, C-3, C-6), 55.1 (2 C, C-8, C-10); MS (ES) m/z (%): 193 [M + 1]^+^, 215 [M + Na] ^+^. Anal. Calcd for C_10_H_12_N_2_O_2_: C, 62.49; H, 6.29; N, 14.57. Found: C, 62.20; H, 6.23; N, 14.57.

#### (1*Z*,1′*Z*)-1,1′-(1,2-Phenylene)bis(*N-tert*-butylmethanimine oxide) (**HBN8**)

Following the general Method B, the reaction of phthalaldehyde (134 mg, 1 mmol) with NaHCO_3_ (252 mg, 3 mmol), Na_2_SO_4_ (568 mg, 4 mmol) and *N*-*tert*-butylhydroxylamine hydrochloride (375 mg, 3 mmol), in EtOH (20 mL), for 5 d , after work-up and purification by column chromatography eluting with AcOEt, gave **HBN8** (157 mg, 57%): mp 130–132 °C; IR (KBr) ν 2,977, 1554, 1,360, 1,195, 1,164, 1,116 cm^−1^; ^1^H NMR (400 MHz, DMSO-d_6_) δ 8.55 (dd, *J* = 5.9, 3.5 Hz, 2 H, H-6, H-3), 7.77 (s, 2 H, H-2, H-7), 7.43 (dd, *J* = 6.0, 3.4 Hz, 2 H, H-5, H-4), 1.52 [s, 18 H, C(CH_3_)_3_]; ^13^C NMR (101 MHz, DMSO-d_6_) δ 130.0 (2 C, C-1, C-2), 129.1 (2 C, C-5, C-4), 128.2 (2 C, C-3, C-6), 127.5 (2 C, C-7, C-9), 71.1 (2 C, C-8, C-10), 28.2 [3 C, C(CH_3_)_3_]; MS (ES) *m/z* (%): 277 [M + 1]^+^, 299 [M + Na]^+^. Anal. Calcd for C_16_H_24_N_2_O_2_: C, 69.53; H, 8.75; N, 10.14. Found: C, 69.51; H, 8.75; N, 10.21.

#### (1*Z*,1′*Z*)-1,1′-(1,2-Phenylene)bis(*N*-benzylmethanimine oxide) (**HBN9**)

Following the general Method B, the reaction of phthalaldehyde (134 mg, 1 mmol) with NaHCO_3_ (252 mg, 3 mmol), Na_2_SO_4_ (568 mg, 4 mmol) and *N*-benzylhydroxylamine hydrochloride (477 mg, 3 mmol) in EtOH (20 mL), for 1 d, after work-up and purification by column chromatography eluting with AcOEt: hexane (3:2), gave **HBN9** (293,5 mg, 85%): mp 152–154 °C; IR (KBr) ν 3,422, 1564, 1,456, 1,149 cm^−1^; ^1^H NMR (500 MHz, DMSO-*d*_6_) δ 8.91 (dd, *J* = 6.0, 3.6 Hz, 2 H, H-4, H-5), 8.27 (s, 2H, H-7, H-9), 7.53–7.50 (m, 4 H, H-3, H-6, C_6_H_5_), 7.42–7.35 (m, 7 H, C_6_H_5_), 5.10 (s, 4 H, H-7, H-9); ^13^C NMR (126 MHz, DMSO-*d*_6_) δ 135.1 (2 C, C_6_H_5_), 130.2 (2 C, C-7, C-9), 129.8 (2 C, C-1, C-2), 129.4 (2 C, C-3, C-6), 128.9 (4 C, C_6_H_5_), 128.8 (4 C, C_6_H_5_), 127.5 (2 C , C-4, C-5), 71.0 (2 C, 8, C-10); MS (ES) *m/z* (%): 350 [M + 1] ^+^, 372 [M + Na]^+^. Anal. Calcd for C_22_H_20_N_2_O_2_: C, 76.72; H, 5.85; N, 8.13. Found: C, 76.46; H, 5.94; N, 8.11.

### Neuroprotection assessment assays

#### Neuroblastoma cell cultures

The human neuroblastomas cell line SH-SY5Y were cultured in Dulbecco's: Ham's F12, 1:1 [vol/vol] containing 3.15 mg/mL glucose, 2.5 mM Glutamax and 0.5 mM sodium pyruvate DMEM/F-12, GlutaMAX™; GIBCO, Life Technologies, Madrid (Spain), 1% Antibiotique-Antimitotic (Gibco; Life Technologies, Madrid, Spain) (containing 100 ui/mL penicillin, 100 mg/mL de streptomycin and 0.25 mg de amphotericine B), 1% gentamicine 15 mg/mL (Sigma-Aldrich, Madrid, España) and 10% Foetal Calf Serum (FCS) (Gibco; Life Technologies, Madrid, Spain) as described^27^. Cultures were seeded into flasks containing supplemented medium and maintained at 37 °C in a humidified atmosphere of 5% CO_2_ and 95% air. Culture media were changed every 2 d. Cells were sub-cultured after partial digestion with 0.25% trypsin–EDTA. For assays, SHSY5Y cells were subcultured in 96 or 48-well plates at a seeding density of 0.50–1 or 2–2.5 × 10^5^ or cells per well, respectively. When the SHSY5Y cells reached 80% confluence, the medium was replaced with fresh medium containing 0.01–1,000 μM compound concentrations or PBS in the controls, as indicated in each assay.

#### Neuroblastoma cell cultures exposure to Oxygen–Glucose deprivation (OGD)

Neuroblastoma cell cultures were exposed to OGD to induce cellular damage (experimental ischemia). Cultured cells were washed and placed in glucose-free Dulbecco’s medium (bubbled with 95% N_2_/5% CO_2_ for 30 min) and maintained in an anaerobic chamber containing a gas mixture of 95% N_2_/5% CO_2_ and humidified at 37 °C at a constant pressure of 0.15 bar. Cells were exposed to OGD for a period of 4 h (OGD 4 h), as indicated. At the end of the OGD period, culture medium was replaced with oxygenated serum-free medium, and cells were placed and maintained in the normoxic incubator for 24 h to recovery (R24h). In the neuroprotection experiments, **HBNs 1–9** and **PBN **(0.01 μM − 1 mM) were added at the beginning of the recovery period (see below). Control cultures in Dulbecco’s medium containing glucose were kept in the normoxic incubator for the same period of time as the OGD (C4h), and then culture medium was replaced with fresh medium and cells were returned to the normoxic incubator until the end of the recovery period (C24h). In each experiment a series of different controls were performed containing the same final % of dimethyl sulfoxide (DMSO) as the samples with the tested compounds (between 0.00001% and 1% of DMSO for the samples with compound concentrations between 0.01 μM and 1,000 μM. In them, cell viability ranged from 100% to 93.5%. This small decrease in cell viability induced by DMSO was taken into account when performing viability and neuroprotection calculations. The control represented in figures is the control of 24 h of incubation with normal culture medium, that is, in the absence of DMSO.

The experimental procedures were blindly performed, assigning a random order to each assayed nitrone. Nitrones were analyzed independently three-five times with different batches of cultures, and each experiment was run in triplicate.

#### Assessment of cell viability

Measurements of cell viability in human SHSY5Y neuroblastoma cells were carried out into 96-well culture plates as described^37^. Briefly, control and treated SH-SY5Y neuroblastoma cells (about 0.75–1 × 105 cells/well) were incubated with the XTT solution (Cell Proliferation Kit II (XTT), Sigma, Aldrich, Madrid) at 0.3 mg/ml final concentration for 2 h in a humidified incubator at 37 °C with 5% CO_2_ and 95% air (v/v) and the soluble orange formazan dye formed was spectrophotometrically quantified, using a Biotek Power-Wave XS spectrophotometer microplate-reader at 450 nm (reference 650 nm). All XTT assays were performed in triplicate in cells of at least three different cell batches. Control cells treated with DMEM alone were regarded as 100% viability. Controls containing different DMSO concentrations (0.001–1% DMSO) were performed in all assays.

#### Measurement of LDH activity

For these assays, cultured neuroblastoma cells grown in 96-well culture dishes at a density of 1.5 × 105 cells/well were used. LDH activity was measured as the rate of decrease of the absorbance at 340 nm, resulting from the oxidation of NADH to NAD + as described^38^. Data are given as the percentage of LDH release with respect to the total LDH content (LDH in the culture medium and LDH inside the cells).

#### Analysis of caspase-3 activity

For these assays, cultured neuroblastoma cells grown in 48-well culture dishes, at a density of 2.5 × 10^5^ cells/well, were used. After OGD treatment, cells were treated with different nitrones or indicated positive controls at 1 − 500 μM concentrations and subjected to 24 h reperfusion. Attached cells were lysed at 4 °C in a lysis medium containing 5 mM Tris/HCl (pH 8.0), 20 mM ethylenediaminetetraacetic acid, and 0.5% Triton X-100 and centrifuged at 13.000*g* for 10 min. The activity of caspase-3 was measured using the fluorogenic substrate peptide DEVD-amc (66081; BD Biosciences PharMingen), as described^38,29^. Proteins were measured by the Bradford assay. Results were expressed as arbitrary fluorescence units [(AFU)/μg protein/h].

#### Measurement of ROS formation

SHSY5Y human neuroblastoma cells (2 × 10^5^ cells/well) were exposed to OGD for a period of 4 h (OGD4h). At the end of the OGD period, the culture medium was replaced with oxygenated Dulbecco’s modified Eagle’s medium containing glucose and 10% fetal calf serum. Cells were treated in the absence (controls) or presence of indicated concentrations of nitrones or different known neuroprotective agents and maintained at 37 °C in a normoxic incubator for 3 h for recovery. At the end of this period, 20 μM DHE (HEt; Molecular Probes) was added and fluorescence was recorded every 15 − 30 s during a 15 min period, using an excitation filter of 535 nm and an emission filter of 635 nm in a spectrofluorimeter (Bio-Tek FL 600) as previously described^34^. Linear regression of fluorescence data [expressed as arbitrary fluorescence units (AFU)] was calculated for each condition, and the slopes (a) of the best fitting lines (y = ax) were considered as an index of O_2_^·−^ production. SNP was used as a positive control of superoxide production^37^.

#### Statistical analysis

Data were expressed as mean ± SEM of results obtained from at least three independent experiments from different cultures, each of which was performed in triplicate. Statistical comparisons between the different experimental conditions were performed using one-way analysis of variance (ANOVA), followed by Holm–Sidak’s post-test when the analysis of variance was significant. A P value < 0.05 was considered statistically significant. Concentration–response curves for the estimation of EC_50_ and maximal activities values were calculated by a weighted nonlinear regression of minimum squares using four parameters logistic curves (f1 = min + (max–min)/(1 + (x/EC_50_)^(-Hillslope), by using the program SigmaPlot v.11 (Systat Software INC., 2012). Lineal correlation analysis (straight line equations, correlation coefficients (r) and statistical significances of regression line equations) were performed by the Pearson Product Moment Correlation Test, carried out with SigmaPlot v.11.

#### pMCAO stoke model

To study of the effects of **HBN6** on stroke recovery, administration of the vehicle and **HBN6** was performed intraperitoneally, to 8­week­old male C57BL/6 J mice (Harlan) weighing 25–30 g. All procedures with animals were carried out under a protocol approved by the Ethical Committee of the Spanish National Research Council (CSIC), and recommendations of the European Council. A special effort was made to keep to a minimum necessary the number of animals to achieve adequate significance. For surgery, anaesthesia induction was carried out with 3% isoflurane (in 70% N_2_O, 30% O_2_), followed by 2% isoflurane for maintenance during stroke procedure. Rectal temperature was maintained at 36.5 °C with the use of a heating pad. The frontal branch of the MCA was, after craniotomy, exposed and occluded permanently by suture ligation as previously reported, with modifications^33^. The permanent occlusion involved exclusively the frontal branch of the middle cerebral artery, the stem of this artery remaining untie. This procedure yielded a smaller infarct size than that determined by ligature of the arterial stem, allowing a better assessment of final infarct volume among vehicle control and **HBN6** treated groups, and also reducing the sample (n) size. To ensure a complete artery occlusion during surgery, cortical blood flow was monitored by non-invasive laser Doppler flowmetry, as a quality control, with the aid of a Perimed equipment (PeriFlux System 5,000 Stroke Model Monitor, Perimed, Järfälla, Sweden). The study was exclusively performed in animals that showed post-ligature a drop of blood flow of at least 65%. Animals subjected to surgery for longer than 15 min were excluded of the study. Physiological parameters were maintained as previously reported^33^. Experiments were performed in each of the following groups: (a) sham operated (n = 6 animals); (b) pMCAO vehicle control group (saline buffer containing 29% dimethyl sulfoxide, DMSO) (n = 6), and (c) pMCAO **HBN6** treated group (100 mg/kg **HBN6** dissolved in vehicle) (n = 6). Drug administration was 15 min after arterial ligature. Determination of infarct size in vehicle and **HBN6** treated mice was performed by means of the 2,3,5-triphenyltetrazolium chloride (TTC) staining procedure of sequential coronal 1 mm-thick brain slices obtained from the operated animals with the aid of a Brain Matrix (WPI, UK) as reported previously^33^. The experiments compared infarct volume outcome between group’s b and c. Sham operated control group (a) showed with certainty that stroke was not due to the surgical pre-occlusive procedure. Infarct volumes, shown in mm^3^, were obtained integrating infarcted areas by counting pixels contained within the regions of interest. Each side of the coronal sections was sampled. Images were taken with the aid of a digital camera (Pentax Optio S7) that provided good resolution of infarct boundaries. With the use of the free software ImageJ 1.33u software (National Institutes of Health, Bethesda, MD), acquired images were analyzed. Student two-sample t-test was carried out to determine the statistical significance of differences of infarct values between the vehicle and the **HBN6** treated mice. P value < 0.05 was considered significant.

### Antioxidant activity tests of HBNs 5, 6, 9, and PBN

#### Estimation of Lipophilicity as Clog P

Bioloom of Biobyte Corp was used for the theoretical calculation of lipophilicity as Clog *P* values (BioByte Home Page. Available online: https://www.biobyte.com).

#### Materials and methods

Nordihydroguaiaretic acid (**NDGA**), Trolox, 2,2′-azobis(2-amidinopropane) dihydrochloride (AAPH), 2,2′-Azino-bis(3-ethylbenzthiazoline-6-sulfonic acid) (ABTS)Soybean LOX linoleic acid sodium salt were purchased from the Aldrich Chemical Co. Milwaukee, WI, (USA). Phosphate buffer (0.1 M and pH 7.4) was prepared mixing an aqueous KH_2_PO_4_ solution (50 mL, 0.2 M), and an aqueous of NaOH solution (78 mL, 0.1 M); the pH (7.4) was adjusted by adding a solution of KH_2_PO_4_ or NaOH). For the in vitro tests a Lambda 20 (Perkin–Elmer-PharmaSpec 1,700) UV–Vis double beam spectrophotometer was used.

### Inhibition of linoleic acid peroxidation^39^

For initiating the free radical, 2,2′-azobis(2-amidinopropane) dihydrochloride (AAPH) is used. The final solution in the UV cuvette consisted of ten microliters of the 16 mM linoleate sodium dispersion 0.93 mL of 0.05 M phosphate buffer, pH 7.4, thermostatted at 37 °C. 50 μL of 40 mM AAPH solution was added as a free radical initiator at 37 °C under air and 10 μL of the tested compounds.

### Inhibition of soybean lipoxygenase^14^

The oxidation of linoleic acid sodium salt results in a conjugated diene hydroperoxide. The reaction is monitored at 234 nm. Soybean lipoxygenase inhibition study in vitro. In vitro study was evaluated as reported previously^14^. The tested compounds (several concentrations 1–100 µM, from the stock solution 10 mM were used for the determination of IC_50_) dissolved in DMSO were incubated at room temperature with sodium linoleate (0.1 mM) and 0.2 mL of enzyme solution (1/9 × 10^–4^ w/v in saline). The conversion of sodium linoleate to 13-hydroperoxylinoleic acid at 234 nm was recorded and compared with the appropriate standard inhibitor NDGA (IC_50_ 0.45 μM and 93% at 100 μM).

### Hydroxyl radicals scavenging activity^39^

The hydroxyl radicals were produced by the Fe^3+^/ascorbic acid system. EDTA (0.1 mM), Fe 3 + (167 μM), DMSO (33 mM) in phosphate buffer (50 mM, pH 7.4), the tested compounds (0.1 mM) and ascorbic acid (10 mM) were mixed in test tubes. The solutions were incubated at 37 °C for 30 min. The reaction was stopped by CCl_3_COOH (17% *w/v*) and the % scavenging activity of the tested compounds for hydroxyl radicals was given.

## Supplementary information

Supplementary information
