# Supplementary material for: Synthesis, antioxidant properties and neuroprotection of α-phenyl-tert-butylnitrone derived HomoBisNitrones in in vitro and in vivo ischemia models
Source: Sci Rep. 2020 Aug 25;10:14150. doi: 10.1038/s41598-020-70690-y (PMC7447640; doi:10.1038/s41598-020-70690-y)
Supplement: Supplementary file 1 — Supplementary information [file 41598_2020_70690_MOESM1_ESM.docx]

**Supplementary Information**

## Synthesis, Antioxidant Properties and Neuroprotection of α-Phenyl-*tert*-butylnitrone Derived *HomoBisNitrones* in *in vitro* and *in vivo* Ischemia Models

Beatriz Chamorro^1,2,♯^, Daniel Diez-Iriepa^3,4,♯^, Belén Merás-Sáiz^3^, Mourad Chioua^3^, David García-Vieira^3^, Isabel Iriepa^4,5^, Dimitra Hadjipavlou-Litina,^6^ Francisco López-Muñoz,^2,7^ Ricardo Martínez-Murillo^8,^*, Daniel Gonzàlez-Nieto^9^, Israel Fernández^10,^*, José Marco-Contelles^3,^*, María Jesús Oset-Gasque ^1,11,^*

^1^Department of Biochemistry and Molecular Biology, Faculty of Pharmacy, Complutense University of Madrid, 28040 Madrid, Spain

^2^Faculty of Health, Camilo José Cela University of Madrid (UCJC). Spain

^3^Laboratory of Medicinal Chemistry^,^ Institute of Organic Chemistry (CSIC), Juan de la Cierva 3, 28006-Madrid, Spain

^4^Department of Organic Chemistry and Inorganic Chemistry, Alcalá University, 28805 Alcalá de Henares, Madrid, Spain

^5^Institute of Chemical Research Andrés M. del Río, Alcalá University, 28805-Alcalá de Henares, Madrid, Spain

^6^Department of Pharmaceutical Chemistry, School of Pharmacy, Faculty of Health Sciences, Aristotle University of Thessaloniki, Thessaloniki 54124, Greece

^7^Neuropsychopharmacology Unit, “Hospital 12 de Octubre” Research Institute, Madrid, Spain

^8^Neurovascular Research Group, Department of Translational Neurobiology, Cajal Institute (CSIC), Madrid, Spain

^9^Center for Biomedical Technology (CTB), Universidad Politécnica de Madrid, Madrid, Spain; Biomedical Research Networking Center in Bioengineering Biomaterials and Nanomedicine (CIBER-BBN). Madrid, Spain

^10^Departamento de Química Orgánica I and Centro de Innovación en Química Avanzada (ORFEO-CINQA), Facultad de Ciencias Químicas, Complutense University of Madrid, 28040-Madrid, Spain

^11^Instituto de Investigación en Neuroquímica, Universidad Complutense de Madrid. 28040 Madrid, Spain

**CONTENTS**

**1. NMR spectra of HBNs 1-9……………………………………………………..S2-S10**

**2. Computational details…………………………………………………………………S11-S30**

**3. ADME analysis………………………………………………………………………...S31-S32**

**1. NMR spectra of HBNs 1-9**

**^1^H and ^13^C NMR spectra of HBN1**

**^1^H and ^13^C NMR spectra of HBN2**

**^1^H and ^13^C NMR spectra of HBN3**

**^1^H and ^13^C NMR spectra of HBN4**

**^1^H and ^13^C NMR spectra of HBN5**

**^1^H and ^13^C NMR spectra of HBN6**

**^1^H and ^13^C NMR spectra of HBN7**

**^1^H and ^13^C NMR spectra of HBN8**

**^1^H and ^13^C NMR spectra of HBN9**

**2. Computational details**

All the calculations reported in this paper were performed with the Gaussian 09 suite of programs.^^[[1]](#footnote-1)^^ Electron correlation was partially taken into account using the hybrid functional usually denoted as B3LYP^^[[2]](#footnote-2)^^ in conjunction with the D3 dispersion correction suggested by Grimme et al.^^[[3]](#footnote-3)^^ using the standard double-ζ quality def2-SVP^^[[4]](#footnote-4)^^ basis set for all atoms. Geometries were fully optimized without any geometry or symmetry constraints. Reactants and products were characterized by frequency calculations,^^[[5]](#footnote-5)^^ and have positive definite Hessian matrices. Transition structures (TS’s) show only one negative eigenvalue in their diagonalized force constant matrices, and their associated eigenvectors were confirmed to correspond to the motion along the reaction coordinate under consideration using the Intrinsic Reaction Coordinate (IRC) method.^^[[6]](#footnote-6)^^ Non-covalent interactions were visualized by means of the NCIPLOT method.^[[7]](#footnote-7)^Cartesian coordinates (in Å) and free energies (in a. u.) of all the stationary points discussed in the text. All calculations have been performed at the B3LYP-D3/def2-SVP.

**HBN6:** E = -1109.139589

C 1.153656000 -0.671630000 -0.434494000

C 1.141099000 -2.086997000 -0.429545000

C 0.000087000 -2.765011000 0.000440000

C -1.140900000 -2.086752000 0.430103000

C -1.153405000 -0.671385000 0.434378000

C 0.000137000 0.009782000 -0.000221000

H 2.028479000 -2.619562000 -0.763071000

H 0.000069000 -3.858185000 0.000703000

H -2.028301000 -2.619127000 0.763883000

H 0.000150000 1.104465000 -0.000492000

C -2.283712000 0.135901000 0.856901000

C 2.283981000 0.135427000 -0.857409000

C -4.514262000 0.681695000 1.644558000

H -4.944083000 0.300006000 2.581371000

H -4.055019000 1.660574000 1.834524000

C 4.514601000 0.680876000 -1.645049000

H 4.944646000 0.298648000 -2.581527000

H 4.055332000 1.659619000 -1.835669000

O -3.783646000 -1.517747000 1.442590000

O 3.783978000 -1.518484000 -1.442188000

N -3.444197000 -0.308044000 1.302305000

N 3.444476000 -0.308732000 -1.302526000

H -2.194093000 1.220996000 0.805868000

H 2.194358000 1.220555000 -0.806972000

C -5.554549000 0.770694000 0.553128000

C -5.744085000 1.968063000 -0.150721000

C -6.333027000 -0.350306000 0.221279000

C -6.700764000 2.051686000 -1.167390000

H -5.140899000 2.846029000 0.099210000

C -7.281569000 -0.268089000 -0.799830000

H -6.166497000 -1.285827000 0.757702000

C -7.470502000 0.932336000 -1.494485000

H -6.841914000 2.992451000 -1.705614000

H -7.879953000 -1.146689000 -1.054285000

H -8.217740000 0.994203000 -2.289827000

C 5.554552000 0.770501000 -0.553354000

C 5.743783000 1.968202000 0.149991000

C 6.333012000 -0.350305000 -0.220750000

C 6.700134000 2.052359000 1.166927000

H 5.140632000 2.846014000 -0.100567000

C 7.281231000 -0.267544000 0.800612000

H 6.166758000 -1.286076000 -0.756824000

C 7.469840000 0.933215000 1.494783000

H 6.841045000 2.993374000 1.704774000

H 7.879620000 -1.145965000 1.055668000

H 8.216783000 0.995484000 2.290373000

**INT0·:** E = -1184.824718

C -1.179959000 1.389109000 -0.155786000

C -1.484311000 2.740927000 0.124818000

C -0.530511000 3.551160000 0.746429000

C 0.718614000 3.047037000 1.095316000

C 1.048541000 1.696813000 0.825278000

C 0.088393000 0.881915000 0.198301000

H -2.463456000 3.126622000 -0.149907000

H -0.771924000 4.595175000 0.961059000

H 1.451824000 3.696562000 1.581746000

H 0.348125000 -0.154154000 -0.009094000

C 2.371317000 1.245699000 1.218680000

C -2.096188000 0.459312000 -0.791355000

C 4.281122000 -0.233901000 1.509912000

H 4.619229000 0.604878000 2.132111000

H 4.229332000 -1.146230000 2.118689000

C -4.142714000 -0.383306000 -1.789342000

H -4.613954000 0.067196000 -2.674114000

H -3.476721000 -1.194480000 -2.110483000

O 2.263864000 -0.955408000 0.557602000

O -3.916923000 1.835799000 -1.125188000

N 2.871674000 0.037667000 1.082815000

N -3.321350000 0.724505000 -1.204152000

H 3.043623000 1.970166000 1.678610000

H -1.763814000 -0.566620000 -0.946549000

C 5.175703000 -0.431518000 0.310738000

C 5.392854000 -1.715318000 -0.209003000

C 5.760637000 0.675379000 -0.321654000

C 6.188446000 -1.886356000 -1.345251000

H 4.936244000 -2.573815000 0.287796000

C 6.555127000 0.504122000 -1.457441000

H 5.594208000 1.680339000 0.078174000

C 6.769006000 -0.779258000 -1.971743000

H 6.355365000 -2.890633000 -1.742793000

H 7.009798000 1.372392000 -1.941065000

H 7.391418000 -0.915615000 -2.859887000

C -5.172907000 -0.878901000 -0.803288000

C -5.079790000 -2.171413000 -0.268820000

C -6.221300000 -0.039812000 -0.392117000

C -6.023217000 -2.626796000 0.657388000

H -4.263714000 -2.830340000 -0.579704000

C -7.158254000 -0.492847000 0.538493000

H -6.276136000 0.973512000 -0.793191000

C -7.063816000 -1.786976000 1.063254000

H -5.942340000 -3.638202000 1.063600000

H -7.969380000 0.167674000 0.855587000

H -7.801897000 -2.139975000 1.788113000

O 3.105858000 -3.330774000 1.599661000

H 2.708752000 -2.527021000 1.157212000

**TS1:** E = -1184.817354

C -1.112716000 1.242346000 -0.520329000

C -1.419369000 2.621814000 -0.556794000

C -0.456459000 3.556216000 -0.166523000

C 0.804617000 3.149429000 0.257321000

C 1.136054000 1.774015000 0.307439000

C 0.165354000 0.833385000 -0.083705000

H -2.407128000 2.931088000 -0.890672000

H -0.699890000 4.621144000 -0.196206000

H 1.546106000 3.894999000 0.557097000

H 0.426162000 -0.222570000 -0.049745000

C 2.474670000 1.431574000 0.749579000

C -2.035122000 0.189841000 -0.907183000

C 4.380401000 0.049136000 1.374481000

H 4.781764000 1.032278000 1.649334000

H 4.287356000 -0.555235000 2.286540000

C -4.084206000 -0.859975000 -1.679989000

H -4.542166000 -0.632526000 -2.652880000

H -3.421345000 -1.727868000 -1.789041000

O 2.322942000 -0.865763000 0.764993000

O -3.855632000 1.451708000 -1.551224000

N 2.968710000 0.219496000 0.913032000

N -3.261352000 0.352215000 -1.367372000

H 3.169258000 2.243398000 0.962529000

H -1.704760000 -0.844433000 -0.816655000

C 5.224849000 -0.618953000 0.319653000

C 5.053771000 -1.982667000 0.034000000

C 6.168723000 0.119091000 -0.408271000

C 5.817040000 -2.593514000 -0.962775000

H 4.304048000 -2.549962000 0.587505000

C 6.937720000 -0.493908000 -1.401998000

H 6.307346000 1.183023000 -0.194072000

C 6.761740000 -1.852004000 -1.681325000

H 5.675949000 -3.655469000 -1.179624000

H 7.674306000 0.090837000 -1.958802000

H 7.361626000 -2.333709000 -2.457782000

C -5.129802000 -1.100814000 -0.618100000

C -5.048245000 -2.223868000 0.216838000

C -6.181712000 -0.187795000 -0.439479000

C -6.005758000 -2.439791000 1.212801000

H -4.229907000 -2.938441000 0.087234000

C -7.132803000 -0.400285000 0.560283000

H -6.228731000 0.696845000 -1.076588000

C -7.049400000 -1.526832000 1.386598000

H -5.933580000 -3.321384000 1.854762000

H -7.946422000 0.317096000 0.694923000

H -7.798326000 -1.692539000 2.165340000

O 2.044073000 0.367533000 3.399118000

H 1.719689000 -0.285009000 2.738953000

**TS2:** E = -1184.819000

C 0.977966000 1.188884000 0.464986000

C 1.286015000 2.560721000 0.389784000

C 0.336432000 3.460437000 -0.108028000

C -0.915373000 3.024400000 -0.536277000

C -1.258259000 1.656686000 -0.472540000

C -0.300209000 0.739666000 0.030457000

H 2.266756000 2.897458000 0.717655000

H 0.585675000 4.522949000 -0.165316000

H -1.638165000 3.745005000 -0.928651000

H -0.617156000 -0.276845000 0.244184000

C -2.568973000 1.269322000 -0.942284000

C 1.894328000 0.162424000 0.901681000

C -4.409536000 -0.172606000 -1.607680000

H -4.780797000 0.753680000 -2.065232000

H -4.320506000 -0.945103000 -2.384535000

C 3.954204000 -0.841389000 1.708280000

H 4.376097000 -0.625672000 2.699964000

H 3.313625000 -1.730072000 1.774030000

O -2.350559000 -1.031384000 -0.936138000

O 3.663466000 1.465250000 1.606598000

N -3.009611000 0.042455000 -1.136713000

N 3.105732000 0.352240000 1.395500000

H -3.277743000 2.057375000 -1.197948000

H 1.598612000 -0.876941000 0.769182000

C -5.308413000 -0.621473000 -0.479525000

C -5.097857000 -1.861742000 0.144594000

C -6.348651000 0.203757000 -0.030665000

C -5.917736000 -2.263397000 1.200801000

H -4.273949000 -2.491975000 -0.194365000

C -7.174171000 -0.203016000 1.022268000

H -6.518672000 1.172640000 -0.509559000

C -6.958735000 -1.437470000 1.640520000

H -5.745376000 -3.228989000 1.683084000

H -7.984875000 0.447280000 1.360436000

H -7.601742000 -1.757249000 2.464502000

C 5.037366000 -1.021134000 0.672689000

C 4.983889000 -2.088503000 -0.234056000

C 6.090833000 -0.097148000 0.584400000

C 5.972435000 -2.240118000 -1.211029000

H 4.160853000 -2.806897000 -0.179147000

C 7.073949000 -0.245475000 -0.395752000

H 6.113994000 0.747347000 1.275172000

C 7.019080000 -1.317786000 -1.293496000

H 5.921061000 -3.077696000 -1.911217000

H 7.888907000 0.480133000 -0.459914000

H 7.791989000 -1.432927000 -2.057839000

O 0.241642000 -0.266764000 -1.874278000

H -0.579337000 -0.803418000 -1.810239000

**TS3:** E= -1184.814833

C -1.240024000 0.994249000 -0.558672000

C -1.438536000 2.370099000 -0.817146000

C -0.408403000 3.279086000 -0.560354000

C 0.815246000 2.850227000 -0.055951000

C 1.041165000 1.477237000 0.209158000

C 0.001848000 0.562828000 -0.045920000

H -2.397490000 2.696260000 -1.213176000

H -0.569237000 4.341520000 -0.759789000

H 1.610886000 3.574185000 0.139978000

H 0.178301000 -0.490569000 0.164969000

C 2.343956000 1.108683000 0.726124000

C -2.241553000 -0.032204000 -0.786029000

C 4.134403000 -0.327665000 1.489567000

H 4.537065000 0.656417000 1.902381000

H 4.071628000 -1.031128000 2.334842000

C -4.362359000 -1.033002000 -1.416082000

H -4.796214000 -0.931425000 -2.420874000

H -3.769089000 -1.955363000 -1.373112000

O 2.060408000 -1.179162000 0.968835000

O -3.953578000 1.244578000 -1.659472000

N 2.743473000 -0.107595000 1.040925000

N -3.448631000 0.146914000 -1.288510000

H 3.089009000 1.886933000 0.892477000

H -1.994477000 -1.060251000 -0.522737000

C 5.052975000 -0.826121000 0.398892000

C 4.569467000 -1.503925000 -0.731229000

C 6.436875000 -0.622633000 0.535073000

C 5.458351000 -1.960713000 -1.708245000

H 3.497617000 -1.683641000 -0.828842000

C 7.321854000 -1.086911000 -0.441008000

H 6.819313000 -0.086118000 1.407935000

C 6.834336000 -1.756865000 -1.567809000

H 5.069791000 -2.484838000 -2.585176000

H 8.395456000 -0.919357000 -0.322763000

H 7.525073000 -2.116973000 -2.334345000

C -5.430084000 -1.019770000 -0.348669000

C -5.451636000 -2.006870000 0.646478000

C -6.399949000 -0.004276000 -0.329125000

C -6.431100000 -1.988451000 1.644365000

H -4.697436000 -2.799404000 0.641627000

C -7.372674000 0.017537000 0.672154000

H -6.364032000 0.774601000 -1.092359000

C -7.393037000 -0.974894000 1.658978000

H -6.439790000 -2.766055000 2.412350000

H -8.121786000 0.813451000 0.682009000

H -8.158938000 -0.957555000 2.438643000

O 5.237812000 2.082532000 1.876736000

H 5.646599000 1.986608000 0.992943000

**INT1:** E = -1184.888398

C -0.223330000 -0.119293000 0.392910000

C 0.057612000 0.908979000 1.323528000

C 1.211852000 0.843888000 2.103008000

C 2.110661000 -0.216775000 1.965762000

C 1.856591000 -1.231116000 1.034971000

C 0.692250000 -1.186107000 0.271428000

H -0.640385000 1.737797000 1.414813000

H 1.419021000 1.640336000 2.821708000

H 3.023205000 -0.244237000 2.567980000

H 0.498719000 -1.993206000 -0.438493000

C 2.891473000 -2.312441000 0.752166000

C -1.392652000 -0.151195000 -0.465014000

C 4.693572000 -0.808126000 -0.356510000

H 5.251336000 -0.913985000 0.587646000

H 5.396167000 -0.988781000 -1.186187000

C -3.488534000 0.545596000 -1.473178000

H -3.622339000 1.511072000 -1.981223000

H -3.214288000 -0.215456000 -2.215086000

O 3.323665000 -2.298628000 -1.568473000

O -2.386420000 1.852016000 0.105888000

N 3.725569000 -1.897181000 -0.428089000

N -2.331684000 0.772615000 -0.549742000

H 3.583557000 -2.399144000 1.609426000

H -1.530518000 -1.012692000 -1.118059000

C 4.093115000 0.586338000 -0.454673000

C 3.020578000 0.848129000 -1.318434000

C 4.606802000 1.625843000 0.331013000

C 2.458038000 2.124488000 -1.374255000

H 2.612050000 0.037418000 -1.924720000

C 4.054421000 2.908355000 0.262897000

H 5.437863000 1.429466000 1.015315000

C 2.972523000 3.158688000 -0.584884000

H 1.604912000 2.311048000 -2.031111000

H 4.461540000 3.709237000 0.885513000

H 2.525453000 4.154959000 -0.626432000

C -4.729507000 0.143009000 -0.712949000

C -5.281209000 -1.134523000 -0.881447000

C -5.331363000 1.037199000 0.187385000

C -6.422503000 -1.515457000 -0.168880000

H -4.816335000 -1.838944000 -1.577649000

C -6.465975000 0.652883000 0.904383000

H -4.884262000 2.021979000 0.332073000

C -7.016087000 -0.621834000 0.726511000

H -6.845670000 -2.512924000 -0.312274000

H -6.926049000 1.353491000 1.605986000

H -7.907363000 -0.917563000 1.285947000

O 2.342129000 -3.544502000 0.445965000

H 2.371229000 -3.588171000 -0.531807000

**INT2:** E = -1184.864459

C 1.047620000 1.086119000 0.359347000

C 1.209462000 2.466862000 0.237298000

C 0.208015000 3.266617000 -0.340645000

C -0.987789000 2.714229000 -0.819692000

C -1.264477000 1.349116000 -0.713734000

C -0.246782000 0.404861000 -0.077276000

H 2.146356000 2.908846000 0.569723000

H 0.377057000 4.341552000 -0.438929000

H -1.727760000 3.372394000 -1.286113000

H -0.732456000 0.007449000 0.841310000

C -2.516370000 0.884414000 -1.188400000

C 2.027007000 0.190896000 0.850653000

C -4.390526000 -0.620359000 -1.618397000

H -4.664660000 0.151200000 -2.350052000

H -4.316054000 -1.587954000 -2.135269000

C 4.164115000 -0.593870000 1.733243000

H 4.608455000 -0.256222000 2.680493000

H 3.581402000 -1.505966000 1.916044000

O -2.434089000 -1.368820000 -0.597206000

O 3.705184000 1.672945000 1.450244000

N -3.016914000 -0.351731000 -1.121317000

N 3.235663000 0.503899000 1.336914000

H -3.188206000 1.608094000 -1.653342000

H 1.811039000 -0.875565000 0.807145000

C -5.395125000 -0.674865000 -0.489177000

C -5.297877000 -1.668825000 0.498206000

C -6.422369000 0.274926000 -0.405715000

C -6.216105000 -1.706024000 1.549133000

H -4.484438000 -2.394262000 0.440575000

C -7.346222000 0.232400000 0.643578000

H -6.504653000 1.054194000 -1.169386000

C -7.243550000 -0.758193000 1.623595000

H -6.131684000 -2.481448000 2.314871000

H -8.145350000 0.976239000 0.695151000

H -7.963441000 -0.792905000 2.445316000

C 5.223016000 -0.825952000 0.680760000

C 5.237430000 -2.011023000 -0.067209000

C 6.188374000 0.160436000 0.422392000

C 6.206832000 -2.215997000 -1.054033000

H 4.483971000 -2.781232000 0.121865000

C 7.151775000 -0.042046000 -0.567552000

H 6.158538000 1.091892000 0.990562000

C 7.165729000 -1.231202000 -1.305759000

H 6.209358000 -3.145571000 -1.628896000

H 7.897797000 0.732296000 -0.764282000

H 7.923543000 -1.388734000 -2.077628000

O 0.083949000 -0.674155000 -0.928826000

H -0.723516000 -1.234339000 -0.925714000

**INT3:** E = -1108.516809

C 1.248661000 1.551860000 0.335194000

C 1.669222000 2.845759000 -0.054790000

C 0.731380000 3.753399000 -0.559893000

C -0.606984000 3.405893000 -0.685209000

C -1.062174000 2.111001000 -0.302779000

C -0.108755000 1.197511000 0.206357000

H 2.719259000 3.110196000 0.045661000

H 1.060617000 4.751471000 -0.859930000

H -1.322993000 4.131263000 -1.081578000

H -0.455959000 0.208915000 0.497637000

C -2.457956000 1.841415000 -0.463812000

C 2.134732000 0.534846000 0.872932000

C -4.470296000 0.646059000 -0.392683000

H -4.877251000 1.575367000 -0.789778000

C 4.190221000 -0.524229000 1.610950000

H 4.855219000 -0.098596000 2.375565000

H 3.489824000 -1.219165000 2.091985000

O -2.498756000 -0.337913000 0.303481000

O 4.140288000 1.677447000 0.859339000

N -3.114012000 0.670876000 -0.163546000

N 3.431430000 0.655411000 1.086800000

H -3.096870000 2.628769000 -0.861860000

H 1.708731000 -0.434357000 1.130649000

C -5.362120000 -0.448039000 -0.164745000

C -4.988301000 -1.725187000 0.338288000

C -6.735913000 -0.229480000 -0.471578000

C -5.950393000 -2.716333000 0.516581000

H -3.942824000 -1.901767000 0.575926000

C -7.683121000 -1.228260000 -0.287026000

H -7.047756000 0.744554000 -0.859410000

C -7.296411000 -2.481806000 0.209314000

H -5.644258000 -3.691861000 0.903517000

H -8.730845000 -1.034115000 -0.530249000

H -8.040659000 -3.268770000 0.354714000

C 4.971583000 -1.209234000 0.515033000

C 4.663548000 -2.524508000 0.140811000

C 6.003621000 -0.529783000 -0.152881000

C 5.377961000 -3.159128000 -0.880167000

H 3.858755000 -3.060341000 0.652769000

C 6.710337000 -1.161489000 -1.177864000

H 6.224435000 0.501555000 0.127061000

C 6.402355000 -2.477385000 -1.542227000

H 5.130532000 -4.186365000 -1.159703000

H 7.509307000 -0.624105000 -1.695268000

H 6.961370000 -2.970225000 -2.341872000

**Reaction involving PBN**

**PBN:** E = -557.546901

C -1.324411000 0.246163000 -0.000157000

C -1.862451000 -1.061942000 -0.000308000

C -2.223483000 1.339833000 0.000104000

C -3.245852000 -1.250797000 -0.000201000

C -3.601115000 1.140190000 0.000237000

C -4.120850000 -0.160346000 0.000078000

H -1.177383000 -1.906026000 -0.000471000

H -1.827376000 2.359761000 0.000218000

H -3.644954000 -2.268638000 -0.000352000

H -4.274776000 2.001052000 0.000463000

H -5.202155000 -0.320117000 0.000190000

C 0.094665000 0.559937000 -0.000255000

H 0.372970000 1.609093000 -0.000646000

N 1.091166000 -0.304964000 0.000147000

O 0.953663000 -1.562618000 0.000607000

C 2.556070000 0.154335000 -0.000055000

C 3.183467000 -0.445631000 -1.265953000

H 2.728734000 -0.009797000 -2.169652000

H 4.263252000 -0.233978000 -1.283385000

H 3.026716000 -1.531659000 -1.284274000

C 3.183591000 -0.444977000 1.266135000

H 4.263495000 -0.233914000 1.282879000

H 2.729529000 -0.008164000 2.169721000

H 3.026196000 -1.530902000 1.285324000

C 2.712394000 1.676073000 -0.000356000

H 2.274037000 2.139969000 0.896169000

H 3.787236000 1.912420000 -0.000110000

H 2.274871000 2.139563000 -0.897571000

**INT1:** E = -633.292474

C 1.333607000 0.243101000 -0.181418000

C 1.576719000 -1.135541000 -0.107373000

C 2.417385000 1.129825000 -0.159109000

C 2.883614000 -1.618241000 -0.011710000

C 3.726038000 0.646735000 -0.061274000

C 3.963106000 -0.728442000 0.011665000

H 0.736509000 -1.832854000 -0.107140000

H 2.225382000 2.202777000 -0.217077000

H 3.060132000 -2.695099000 0.052606000

H 4.563175000 1.349490000 -0.041856000

H 4.985463000 -1.107220000 0.089685000

C -0.078193000 0.792310000 -0.357805000

H -0.326331000 0.795841000 -1.427064000

N -1.055283000 -0.063199000 0.343661000

O -0.880598000 -0.117299000 1.606929000

C -2.445218000 -0.300028000 -0.169098000

C -3.308093000 0.909598000 0.233048000

H -2.905419000 1.834719000 -0.205482000

H -4.347136000 0.773800000 -0.105873000

H -3.303593000 1.018407000 1.327765000

C -2.956604000 -1.582777000 0.500880000

H -4.002322000 -1.763543000 0.209371000

H -2.353420000 -2.450957000 0.191289000

H -2.898319000 -1.495297000 1.593169000

C -2.429535000 -0.480669000 -1.692302000

H -1.692672000 -1.241375000 -1.995420000

H -3.422105000 -0.819808000 -2.024902000

H -2.211749000 0.456591000 -2.225678000

O -0.212670000 2.123666000 0.070160000

H -0.071429000 2.110756000 1.031244000

**INT2:** E = -633.259104

C 1.171051000 0.481317000 0.004910000

C 1.755909000 -0.915185000 0.220604000

C 2.039940000 1.594834000 -0.137006000

C 3.257360000 -0.931181000 0.281917000

C 3.411607000 1.470296000 -0.049388000

C 4.019700000 0.181409000 0.165523000

H 1.384400000 -1.263072000 1.211805000

H 1.602670000 2.581289000 -0.322680000

H 3.703992000 -1.921273000 0.403705000

H 4.046802000 2.352266000 -0.160811000

H 5.109765000 0.110574000 0.212703000

C -0.213063000 0.720354000 -0.067583000

H -0.535360000 1.740287000 -0.260620000

N -1.211969000 -0.165243000 0.101367000

O -1.017417000 -1.405928000 0.356319000

C -2.680171000 0.217317000 0.009763000

C -3.307055000 -0.137228000 1.366899000

H -2.877785000 0.484626000 2.168302000

H -4.392827000 0.039156000 1.334233000

H -3.120490000 -1.192339000 1.604951000

C -3.279737000 -0.641909000 -1.114429000

H -4.363326000 -0.462876000 -1.184060000

H -2.821844000 -0.388736000 -2.083621000

H -3.103493000 -1.706182000 -0.912015000

C -2.888732000 1.702639000 -0.296490000

H -2.456127000 1.992176000 -1.266045000

H -3.970859000 1.894074000 -0.348871000

H -2.478028000 2.353050000 0.490548000

O 1.346152000 -1.832996000 -0.774335000

H 0.405566000 -2.000903000 -0.561285000

**INT3:** E = -556.888344

C -1.291131000 0.246820000 -0.008410000

C -1.833533000 -1.059357000 -0.004203000

C -2.185719000 1.343925000 -0.000588000

C -3.217490000 -1.243278000 0.007449000

C -3.564030000 1.149188000 0.010521000

C -4.088327000 -0.149479000 0.014705000

H -1.151810000 -1.906219000 -0.010312000

H -1.785796000 2.362324000 -0.003874000

H -3.620342000 -2.259609000 0.010616000

H -4.234611000 2.012409000 0.015876000

H -5.170162000 -0.305305000 0.023470000

C 0.129290000 0.554386000 -0.020059000

H 0.413587000 1.602100000 -0.020907000

N 1.120111000 -0.315482000 -0.032247000

O 0.983109000 -1.571920000 -0.040165000

C 2.591751000 0.143016000 -0.042427000

C 3.193747000 -0.432264000 -1.287432000

H 4.084312000 0.029490000 -1.718385000

H 2.846635000 -1.403659000 -1.640896000

C 3.221043000 -0.484647000 1.222519000

H 4.300826000 -0.276131000 1.242249000

H 2.761837000 -0.057704000 2.129668000

H 3.058453000 -1.569322000 1.218702000

C 2.761120000 1.665832000 -0.008874000

H 2.329477000 2.107686000 0.901687000

H 3.838175000 1.891873000 -0.006347000

H 2.323447000 2.150939000 -0.893709000

**Reaction involving HBN5**

**HBN5:** E = -883.076754

C -1.234525000 0.536496000 0.000020000

C -1.219970000 1.951736000 -0.000038000

C -0.000014000 2.628881000 0.000001000

C 1.219952000 1.951751000 0.000064000

C 1.234527000 0.536509000 0.000057000

C 0.000004000 -0.142195000 0.000051000

H -2.167070000 2.485004000 -0.000160000

H -0.000021000 3.722183000 -0.000021000

H 2.167043000 2.485032000 0.000164000

H 0.000011000 -1.237097000 0.000070000

C 2.436142000 -0.282776000 0.000027000

C -2.436136000 -0.282795000 0.000081000

C 4.872173000 -0.817847000 -0.000026000

C -4.872174000 -0.817843000 0.000000000

O 4.020738000 1.370381000 -0.000029000

O -4.020702000 1.370379000 -0.000037000

N 3.681781000 0.151418000 0.000043000

N -3.681769000 0.151409000 0.000039000

H 2.306181000 -1.360383000 -0.000053000

H -2.306176000 -1.360402000 0.000190000

C -4.454534000 -2.289881000 0.000133000

H -5.366206000 -2.906191000 0.000537000

H -3.876525000 -2.559243000 -0.896847000

H -3.876101000 -2.558858000 0.896966000

C -5.677146000 -0.493310000 -1.266008000

H -6.601836000 -1.089776000 -1.283431000

H -5.933213000 0.573667000 -1.284447000

H -5.093527000 -0.729903000 -2.169775000

C -5.677501000 -0.493361000 1.265735000

H -5.094497000 -0.730539000 2.169758000

H -5.933127000 0.573720000 1.284424000

H -6.602531000 -1.089324000 1.282503000

C 4.454513000 -2.289881000 -0.000462000

H 5.366176000 -2.906203000 -0.000686000

H 3.876241000 -2.559343000 0.896319000

H 3.876334000 -2.558740000 -0.897495000

C 5.677082000 -0.493564000 1.266085000

H 6.601729000 -1.090099000 1.283476000

H 5.933222000 0.573391000 1.284706000

H 5.093387000 -0.730257000 2.169777000

C 5.677565000 -0.493127000 -1.265658000

H 5.094638000 -0.730211000 -2.169756000

H 5.933118000 0.573976000 -1.284175000

H 6.602638000 -1.089021000 -1.282451000

**INT1:** E = -958.824662

C 0.900472000 0.847903000 -0.105940000

C 0.930225000 2.110903000 0.534598000

C -0.230812000 2.879146000 0.608942000

C -1.434180000 2.419350000 0.063265000

C -1.483904000 1.169805000 -0.566615000

C -0.322246000 0.400511000 -0.650030000

H 1.868602000 2.460338000 0.957679000

H -0.196287000 3.855757000 1.098791000

H -2.335670000 3.036190000 0.122850000

H -0.374654000 -0.579187000 -1.132588000

C -2.781764000 0.645696000 -1.174296000

C 2.042699000 -0.039563000 -0.249394000

C -3.609156000 -0.875972000 0.839026000

C 4.396606000 -0.835217000 -0.032457000

O -2.658474000 -1.691554000 -1.176617000

O 3.631139000 1.216643000 0.816953000

N -3.137924000 -0.679134000 -0.565654000

N 3.267129000 0.180904000 0.189437000

H -3.598229000 1.350176000 -0.956959000

H 1.880273000 -0.977256000 -0.771047000

O -2.700057000 0.459507000 -2.545706000

H -2.459712000 -0.483301000 -2.648388000

C -4.609151000 -2.043243000 0.809078000

H -4.961181000 -2.260597000 1.828912000

H -5.480215000 -1.793592000 0.183449000

H -4.133550000 -2.942183000 0.395188000

C -4.297012000 0.393848000 1.352278000

H -3.599243000 1.239398000 1.431549000

H -5.142659000 0.684841000 0.710042000

H -4.693481000 0.198338000 2.359837000

C -2.396523000 -1.231203000 1.719433000

H -1.872349000 -2.099224000 1.294294000

H -1.689709000 -0.391317000 1.779704000

H -2.730893000 -1.482748000 2.737804000

C 4.898672000 -1.216097000 1.367073000

H 4.116250000 -1.750611000 1.928869000

H 5.775874000 -1.875008000 1.281214000

H 5.176538000 -0.313301000 1.925728000

C 3.944761000 -2.084032000 -0.792064000

H 3.167316000 -2.643479000 -0.250137000

H 3.580767000 -1.848663000 -1.803569000

H 4.812684000 -2.751365000 -0.902498000

C 5.478782000 -0.090918000 -0.826494000

H 6.361961000 -0.736189000 -0.947377000

H 5.107902000 0.182299000 -1.827036000

H 5.769553000 0.826015000 -0.298258000

**INT2:** E = -958.801120

C 1.180399000 0.869836000 0.074459000

C 1.344873000 2.254660000 0.046948000

C 0.235276000 3.116042000 -0.016904000

C -1.076192000 2.622549000 -0.061278000

C -1.354243000 1.255028000 -0.005645000

C -0.213270000 0.245781000 0.091712000

H 2.356381000 2.653765000 0.044324000

H 0.401377000 4.195273000 -0.056092000

H -1.906990000 3.331472000 -0.139172000

H -0.333027000 -0.258412000 1.075912000

C -2.717672000 0.855618000 -0.028323000

C 2.224711000 -0.088556000 0.065864000

C -4.708387000 -0.666137000 0.051472000

C 4.569485000 -0.976065000 0.033613000

O -2.495321000 -1.424126000 0.277268000

O 4.045283000 1.313812000 0.107899000

N -3.216400000 -0.374110000 0.101921000

N 3.540919000 0.152894000 0.073137000

H -3.458145000 1.642421000 -0.146937000

H 1.930891000 -1.131004000 0.009516000

O -0.278379000 -0.727576000 -0.933593000

H -1.054651000 -1.276185000 -0.681072000

C -4.917508000 -1.628838000 -1.127617000

H -4.657052000 -1.139617000 -2.079427000

H -4.284413000 -2.517045000 -1.006094000

H -5.972009000 -1.940194000 -1.173333000

C -5.555788000 0.593950000 -0.141076000

H -5.337148000 1.102855000 -1.092062000

H -6.614628000 0.296035000 -0.164443000

H -5.434256000 1.308704000 0.686997000

C -5.062373000 -1.342866000 1.384412000

H -6.117877000 -1.654032000 1.376047000

H -4.426848000 -2.223727000 1.540545000

H -4.911045000 -0.647828000 2.225383000

C 5.409419000 -0.741142000 -1.230355000

H 6.230613000 -1.472566000 -1.275897000

H 4.790600000 -0.855881000 -2.134263000

H 5.829463000 0.273056000 -1.220320000

C 5.423138000 -0.820016000 1.300388000

H 4.813967000 -0.989292000 2.202456000

H 6.242979000 -1.554215000 1.292426000

H 5.845302000 0.192192000 1.348353000

C 3.928414000 -2.364943000 -0.007289000

H 3.306744000 -2.510211000 -0.903210000

H 4.733431000 -3.114787000 -0.036755000

H 3.320341000 -2.568570000 0.886837000

**INT3:** E = -882.419616

C -1.058759000 0.866494000 -0.000938000

C -1.266105000 2.264109000 0.011371000

C -0.164994000 3.125086000 0.011657000

C 1.134331000 2.627561000 -0.000012000

C 1.372080000 1.231185000 -0.012019000

C 0.262251000 0.366765000 -0.011996000

H -2.284783000 2.643304000 0.020038000

H -0.329796000 4.205652000 0.020917000

H 1.981676000 3.319472000 -0.000128000

H 0.449132000 -0.705042000 -0.021366000

C 2.758442000 0.793535000 -0.024674000

C -2.119348000 -0.128556000 -0.004158000

C 4.690134000 -0.787442000 -0.041418000

C -4.441286000 -1.036111000 -0.001128000

O 2.441893000 -1.476312000 -0.031604000

O -3.940662000 1.259499000 0.018503000

N 3.186719000 -0.452871000 -0.031955000

N -3.416878000 0.107129000 0.004559000

H 3.523345000 1.563805000 -0.029866000

H -1.818487000 -1.171131000 -0.015178000

C 5.594568000 0.449725000 -0.033172000

H 5.445582000 1.061779000 0.868794000

H 6.641094000 0.109649000 -0.031924000

H 5.449325000 1.073530000 -0.927232000

C 4.928615000 -1.622498000 1.237695000

H 5.968341000 -1.980946000 1.258833000

H 4.748971000 -1.006721000 2.134731000

H 4.246258000 -2.481180000 1.253238000

C -5.278190000 -0.854843000 1.272602000

H -6.096723000 -1.590343000 1.289593000

H -4.657230000 -1.005208000 2.169961000

H -5.699952000 0.157872000 1.304320000

C -3.797208000 -2.423574000 -0.020535000

H -3.178062000 -2.608202000 0.870178000

H -4.600522000 -3.175759000 -0.024502000

H -3.188379000 -2.587621000 -0.922315000

C -5.294926000 -0.827543000 -1.259494000

H -4.686206000 -0.958946000 -2.168145000

H -6.113976000 -1.562363000 -1.281107000

H -5.716626000 0.185671000 -1.264062000

C 4.923099000 -1.608385000 -1.272673000

H 5.926332000 -1.665235000 -1.699782000

H 4.135720000 -2.280389000 -1.615077000

**3. ADME analysis**

Table 1S. Predicted ADME and molecular properties for compounds **HBN1-9** and **PBN.**

| Nitrone | CNS | MW | SASA | volume | donorHB | accptHB | QPlogPo/w | QPlogS |
| --- | --- | --- | --- | --- | --- | --- | --- | --- |
| **HBN1** | 0 | 192.217 | 453.367 | 724.631 | 0.000 | 2.000 | 2.686 | -3.110 |
| **HBN2** | 0 | 276.378 | 600.361 | 1039.618 | 0.000 | 2.000 | 4.939 | -5.565 |
| **HBN3** | 0 | 344.412 | 682.743 | 1188.993 | 0.000 | 2.000 | 6.247 | -6.806 |
| **HBN4** | 0 | 192.217 | 451.101 | 721.806 | 0.000 | 2.000 | 2.680 | -3.067 |
| **HBN5** | 0 | 276.378 | 595.541 | 1030.859 | 0.000 | 2.000 | 4.906 | -5.479 |
| **HBN6** | 0 | 344.412 | 686.317 | 1190.089 | 0.000 | 2.000 | 6.244 | -6.873 |
| **HBN7** | 0 | 192.217 | 401.680 | 667.298 | 0.000 | 2.000 | 2.490 | -2.131 |
| **HBN8** | 0 | 276.378 | 512.440 | 938.128 | 0.000 | 2.000 | 4.445 | -3.904 |
| **HBN9** | 0 | 344.412 | 632.501 | 1130.892 | 0.000 | 2.000 | 6.048 | -5.853 |
| **PBN** | 1 | 177.246 | 429.378 | 702.961 | 0.000 | 1.000 | 3.450 | -3.342 |

| Nitrone | QPPCaco | QPlogBB | metab | QPlogKhsa | %HOA | PSA | ROF | ROT |
| --- | --- | --- | --- | --- | --- | --- | --- | --- |
| **HBN1** | 1818.235 | -0.376 | 0 | 0.017 | 100.000 | 45.825 | 0 | 0 |
| **HBN2** | 3512.262 | -0.261 | 0 | 0.846 | 100.000 | 39.769 | 0 | 0 |
| **HBN3** | 2525.798 | -0.579 | 2 | 1.195 | 100.000 | 44.047 | 1 | 1 |
| **HBN4** | 1877.838 | -0.360 | 0 | 0.009 | 100.000 | 44.993 | 0 | 0 |
| **HBN5** | 3743.888 | -0.231 | 0 | 0.821 | 100.000 | 38.181 | 0 | 0 |
| **HBN6** | 2445.856 | -0.601 | 2 | 1.198 | 100.000 | 43.368 | 1 | 1 |
| **HBN7** | 2908.684 | -0.120 | 0 | -0.150 | 100.000 | 34.977 | 0 | 0 |
| **HBN8** | 5475.768 | -0.019 | 0 | 0.552 | 100.000 | 25.772 | 0 | 0 |
| **HBN9** | 4122.006 | -0.307 | 2 | 1.026 | 100.000 | 32.949 | 1 | 1 |
| **PBN** | 6116.472 | 0.197 | 0 | 0.264 | 100.000 | 18.991 | 0 | 0 |

CNS: Predicted central nervous system activity on a –2 (inactive) to +2 (active) scale. MW: Molecular weight of the molecule (130.0-725.0). SASA: Total Solvent Accessible Surface Area, in square angstroms, using a probe with a 1.4Å radius (limits 300.0-1000.0). volume: Total solvent-accessible volume, in cubic angstroms, using a probe with a 1.4 Å radius (limits 500.0-2000.0). donorHB: Estimated number of hydrogen bonds that would be accepted by the solute (limits: 0.0-6.0). accptHB: Estimated number of hydrogen bonds that would be donated by the solute (limits: 2.0-20.0). QPlogPo/w: Predicted octanol/water partition coefficient (limits -2.0-6.5). QPlogS: Predicted aqueous solubility. S, in mol/dm^3^, is the concentration of the solute’s saturated solution that is in equilibrium with crystalline solid (limits -6.5-0.5). QPPCaco: Predicted apparent Caco-2 cell permeability in nm/sec. Caco-2 cells is a model for the gut-blood barrier. QikProp predictions are for non-active transport. (< 25 poor, > 500 great). QPlog BB: Predicted brain/blood partition coefficient (limits -3.0–1.2). metab: Number of likely metabolic reactions (limits 1-8). QPlogKhsa: Prediction of binding to human serum albumin (limits -1.5-1.5). HOA: Predicted qualitative Human Oral Absorption on 0 to 100% scale. PSA: Van der Waals surface area of polar nitrogen and oxygen atoms (limits 7.0-200.0). ROF: Number of violations of Lipinski's Rule Of Five. ROT: Number of violations of Jorgensen's rule of three (QPlogS > -5.7, QPCaco > 22 nm/s, number of primary metabolites < 7).

1. (1) Gaussian 09, Revision D.01 (2009) Frisch, M. J., Trucks, G. W., Schlegel, H. B., Scuseria, G. E., Robb, M. A., Cheeseman, J. R., Scalmani, G.; Barone, V.; Mennucci, B., Petersson, G. A., Nakatsuji, H., Caricato, M., Li, X., Hratchian, H. P., Izmaylov, A. F., Bloino, J., Zheng, G., Sonnenberg, J. L., Hada, M., Ehara, M.; Toyota, K.; Fukuda, R.; Hasegawa, J.; Ishida, M.; Nakajima, T.; Honda, Y.; Kitao, O.; Nakai, H., Vreven, T., Montgomery, J. A., Jr., Peralta, J. E., Ogliaro, F., Bearpark, M., Heyd, J. J., Brothers, E., Kudin, K. N., Staroverov, V. N., Kobayashi, R., Normand, J., Raghavachari, K., Rendell, A., Burant, J. C., Iyengar, S. S.; Tomasi, J.; Cossi, M.; Rega, N.; Millam, J. M.; Klene, M.; Knox, J. E.; Cross, J. B.; Bakken, V., Adamo, C., Jaramillo, J., Gomperts, R., Stratmann, R. E., Yazyev, O., Austin, A. J., Cammi, R., Pomelli, C., Ochterski, J. W., Martin, R. L., Morokuma, K., Zakrzewski, V. G., Voth, G. A., Salvador, P., Dannenberg, J. J., Dapprich, S., Daniels, A. D., Farkas, Ö., Foresman, J. B., Ortiz, J. V., Cioslowski, J., and Fox, D. J. Gaussian, Inc., Wallingford CT. [↑](#footnote-ref-1)
2. (2) (a) Becke, A. D. (1993) Density-Functional Thermochemistry. III. The Role of Exact Exchange. *J. Chem. Phys*. *98*, 5648-5652; (b) Lee, C., Yang, W., and Parr, R. G. (1998) *Phys. Rev. B* Development of the Colle-Salvetti Correlation-Energy Formula into a Functional of the Electron Density. *37*, 785-789; (c) Vosko, S. H., Wilk, L., and Nusair, M. (1980) *Can. J. Phys*. Accurate Spin-Dependent Electron Liquid Correlation Energies for Local Spin Density Calculations: A Critical Analysis. *58*, 1200-1211.

   (3) Grimme, S., Antony, J., Ehrlich, S., and Krieg, H. J. (2010) A Consistent and Accurate *ab initio* Parametrization of Density Functional Dispersion Correction (DFT-D) for the 94 Elements H-Pu. *Chem. Phys*. *132*, 154104-154119.

   (4) Weigend, F.. and Ahlrichs, R. (2005) Balanced Basis Sets of Split Valence, Triple Zeta Valence and

   Quadruple Zeta Valence Quality for H to Rn: Design and assessment of accuracy. *Phys. Chem. Chem. Phys*. *7*, 3297-3305.

   (5) McIver, J. W., and Komornicki, A. K. (1972) Structure of Transition States in Organic Reactions. General Theory and an Application to the Cyclobutene-Butadiene Isomerization Using a Semiempirical Molecular Orbital Method. *J. Am. Chem. Soc. 94*, 2625-2633. [↑](#footnote-ref-2)
3. [↑](#footnote-ref-3)
4. [↑](#footnote-ref-4)
5. [↑](#footnote-ref-5)
6. (6) González, C. and Schlegel, H. B. (1990) Reaction Path Following In Mass-Weighted Internal Coordinates. *J. Phys. Chem*. *94*, 5523-5527. [↑](#footnote-ref-6)
7. (7) Johnson, E. R. Keinan, S., Mori-Sánchez, P., Contreras-García, J., Cohen, A. J., and Yang, W. (2010) Revealing Noncovalent Interactions. *J. Am. Chem. Soc*. *13*2, 6498-6506. [↑](#footnote-ref-7)
